# Common mitochondrial deletions in RNA-Seq: evaluation of bulk, single-cell, and spatial transcriptomic datasets

**DOI:** 10.1038/s42003-024-05877-4

**Published:** 2024-02-17

**Authors:** Audrey A. Omidsalar, Carmel G. McCullough, Lili Xu, Stanley Boedijono, Daniel Gerke, Michelle G. Webb, Zarko Manojlovic, Adolfo Sequeira, Mark F. Lew, Marco Santorelli, Geidy E. Serrano, Thomas G. Beach, Agenor Limon, Marquis P. Vawter, Brooke E. Hjelm

**Affiliations:** 1 Department of Translational Genomics, Keck School of Medicine of USC, Los Angeles, CA USA; 2 Department of Psychiatry and Human Behavior, University of California - Irvine (UCI) School of Medicine, Irvine, CA USA; 3 Department of Neurology, Keck School of Medicine of USC, Los Angeles, CA USA; 4 Department of Stem Cell Biology and Regenerative Medicine, Keck School of Medicine of USC, Los Angeles, CA USA; 5 Banner Sun Health Research Institute (BSHRI), Sun City, AZ USA; 6 Mitchell Center for Neurodegenerative Diseases, Department of Neurology, School of Medicine, University of Texas Medical Branch, Galveston, TX USA

**Keywords:** Genomics, Computational biology and bioinformatics, Neural ageing, Neurological disorders, Metabolic disorders

## Abstract

Common mitochondrial DNA (mtDNA) deletions are large structural variants in the mitochondrial genome that accumulate in metabolically active tissues with age and have been investigated in various diseases. We applied the Splice-Break2 pipeline (designed for high-throughput quantification of mtDNA deletions) to human RNA-Seq datasets and describe the methodological considerations for evaluating common deletions in bulk, single-cell, and spatial transcriptomics datasets. A robust evaluation of 1570 samples from 14 RNA-Seq studies showed: (i) the abundance of some common deletions detected in PCR-amplified mtDNA correlates with levels observed in RNA-Seq data; (ii) RNA-Seq library preparation method has a strong effect on deletion detection; (iii) deletions had a significant, positive correlation with age in brain and muscle; (iv) deletions were enriched in cortical grey matter, specifically in layers 3 and 5; and (v) brain regions with dopaminergic neurons (i.e., substantia nigra, ventral tegmental area, and caudate nucleus) had remarkable enrichment of common mtDNA deletions.

## Abstract (web summary)

A methods application study demonstrates that the Splice-Break2 pipeline can detect large mitochondrial DNA deletions in RNA-Seq data. Analyses includes GTEx and GEO datasets and demonstrate differences across tissues, brain regions, and age.

## Introduction

Mitochondrial DNA (mtDNA) deletions are large structural variants in the mitochondrial genome where a large piece of DNA (often several kilobases or more) is missing^1–6^. These mtDNA molecules may or may not have the ability to replicate and can lead to metabolic impairment by disrupting regions that code for proteins, ribosomal and/or transfer RNAs essential for the oxidative phosphorylation (OXPHOS) pathway^3,7–9^. The identification of mtDNA deletion breakpoints and the relative quantification of deleted vs. wild-type mitochondrial genomes have traditionally relied on several approaches, including Southern Blot analysis, Sanger sequencing, and quantitative polymerase chain reaction (qPCR)^10–12^. Recently, however, several bioinformatics programs and pipelines have been described to identify mtDNA deletions with high accuracy using next-generation sequencing (NGS) data^13–17^. Our group developed the Splice-Break pipeline for this purpose, and we previously demonstrated its accuracy and applications to human disease and aging research when used on Illumina sequencing data derived from NGS libraries prepared using mitochondrial-enriched, long-range PCR products as the DNA input^13^. While PCR amplification of the mitochondrial genome in one or more large fragments is a common approach used by many mitochondrial research groups, it still represents a niche method, and such sequencing data is limited. With the more widespread availability of RNA-sequencing (RNA-Seq) data from a variety of tissues and phenotypes, however, it is of great interest to determine if these datasets can be utilized as a resource for mtDNA deletion investigations.

In this study, we demonstrate how Splice-Break2^13^ may be utilized to evaluate mtDNA deletions from RNA-Seq data. We describe a robust evaluation of 30 common mtDNA deletions in 1570 human samples from 14 RNA-Seq studies^18–29^, including 1107 samples across 11 tissues from the Genotype-Tissue Expression (GTEx) Project^29^. Studies analyzed include both publicly available and newly presented datasets and cover a variety of methods including those that utilized bulk RNA-Seq (polyA/non-ribosomal depletion vs. ribosomal depletion), laser capture microdissection (LCM) RNA-Seq, spatial transcriptomics, and single-cell RNA sequencing (scRNA-Seq) library preparation methods. These 14 studies include 1570 samples that vary in age, diagnosis and tissue, including: (1) age ranges from prenatal to 96 years; (2) diagnoses of Parkinson’s Disease (PD)^18,22,24^, Alzheimer’s Disease (AD)^19^, schizophrenia (SCZ)^20,21^, major depressive disorder (MDD)^21^, bipolar disorder (BD)^21^, and healthy controls (CTRL); and (3) tissues of several brain regions^18–21,24–26,28,29^, skeletal muscle^22,23,29^, liver^29^, blood^29^, and the pancreas^27^.

Large mtDNA deletions are often associated with short repeat sequences proximal to the 5′ and 3′ breakpoints; these repeats lead to an incorrect joining of two distal regions of the mitochondrial genome, either through strand-displacement during mtDNA replication or homologous end-joining during DNA repair processes^30–33^. Our previous study evaluating mtDNA deletions from brain and blood samples found that all the “Top 30” most frequent mtDNA deletions (which were detected in 69% or more of all samples) were associated with (perfect/imperfect) repeat sequences of 8–22 base pairs in length. These 30 deletions were previously validated by Sanger sequencing and include 12 breakpoints that had been previously described in patients with mitochondrial deletion disorders^13,34^. Three such examples are the 6335–13999, 7816–14807, and 8471–13449 deletions, which represent 5′-3′ positions 6341–14005, 7814–14805, and 8482–13460, respectively, when adjusted to the first repeat base^13,33,35–39^. These deleted molecules have been associated with a number of rare diseases including (but not limited to) Kearns-Sayre Syndrome (KSS)^36,37^, chronic progressive external ophthalmoplegia (CPEO) with or without an associated *POLG* mutation^37,40,41^, and diffuse leukodystrophy^39^; moreover, they have been found to be enriched in metabolically-active, somatic tissues and have been investigated in pathogenic phenotypes associated with cellular aging (e.g., PD, AD)^42–44^.

MtDNA sequences present in RNA-Seq datasets are likely derived from real, transcribed RNA molecules, but may also be attributed to DNA contamination or “off-target” effects^26,45,46^. The amount of mitochondrial gene transcripts detected in RNA-Seq studies can also be highly variable due to overall RNA quality and library preparation procedures^26,45–51^. Due to these concerns, many RNA-Seq and ATAC-Seq bioinformatics pipelines remove mitochondrial reads prior to genome alignment and/or transcript quantification^26,45–51^. As such, our first objective was to determine which subset or species of mtDNA deletions demonstrated a significant and positive correlation between RNA-Seq and the aforementioned DNA sequencing processes that utilize long-range PCR amplification of the mitochondrial genome. We PCR-amplified mtDNA from the dorsolateral prefrontal cortex (DLPFC) and processed the NGS data through the traditional Splice-Break2 pipeline^13^, and compared the deletion read %’s to mRNA-Seq data that was previously published by Zeppillo et al. using the same tissue (GSE224683)^20^. Next, we evaluated 14 RNA-Seq studies^18–29^ to determine how methodological differences in library preparation and sequencing depth affected our ability to detect mitochondrial reads and common mtDNA deletions. Lastly, taking our library preparation observations into account, we evaluated 11 of the 14 RNA-Seq datasets derived from human brain, muscle tissue, liver and blood, and examined if these common deletions significantly correlated with age^20–23,29^, were enriched in disease phenotypes or brain regions^18–22,24,25,29^, or had differential levels in cortical grey matter layers (I-VI) compared to white matter^26^. Cortical layer evaluations were performed using two spatial transcriptomics datasets, including one dataset of middle temporal gyrus (MTG) that is newly presented here in this study^26^ (GSE226663).

Here, we describe the methodological considerations for applying the Splice-Break2 pipeline to RNA-Seq datasets, and present evidence that somatic aging effects, tissue specificity, and metabolic dysfunction can be studied with this approach. Given the wealth of both publicly available and restricted RNA-Seq datasets, this strategy may expedite investigations of common mtDNA deletions, especially for cases where the affected human tissue is not readily available for additional sequencing studies (e.g., rare diseases). Moreover, RNA-Seq studies are already designed to appreciate factors such as tissue and cellular specificity, environmental context, aging and drug exposure, whereas germline DNA studies (e.g., whole genome sequencing (WGS)) often use DNA derived from saliva or blood, which may not accurately recapitulate the somatic structural variation present in metabolically-active tissues or cells^52,53^. Taken together, we believe this study will open a new door to study common mtDNA deletions and their role in human health and disease.

## Methods

### Samples

We evaluated 1570 samples from 14 RNA-Seq datasets. Thirteen of these datasets are provided on the National Center for Biotechnology Information’s (NCBI) Gene Expression Omnibus (GEO) website or are otherwise publicly available- we refer to these datasets collectively as “GEO+”^18–28^. This consisted of 7 bulk sequencing with and without ribosomal depletion (GSE226663, GSE114517, GSE159699, GSE224683, GSE140089, GSE164471, and the Stanley Neuropathology Consortium dataset: http://sncid.stanleyresearch.org), 2 LCM RNA-Seq (GSE114918 and GSE166024), 2 spatial transcriptomics (GSE226663 and SpatialLIBD dataset available at Globus endpoint “jhpce#HumanPilot10x”), and 2 single-cell sequencing datasets (GSE81547 and GSE67835). The last dataset came from the Genotype-Tissue Expression Consortium (GTEx)^29^, where we analyzed bulk sequencing samples from 11 human tissues (dbGaP phs000424.vN.pN). All described studies obtained consent from living donors and/or next-of-kin for postmortem tissues, along with necessary approvals from Institutional Review Boards (IRBs) and/or ethics committees.

The 30 samples derived from mitochondrial-enriched, long-range PCR products as the DNA input are available through dbGaP (phs002395.v1.p1) as part of a larger investigation of blood and brain-derived mtDNA^13,54^. DNA sequencing was performed on mitochondrial enriched PCR-amplicons fragmented by sonication and prepared for NGS using a whole-genome library preparation kit^13,55^. Briefly, ~50 mg of frozen brain tissue was homogenized manually with a pestel; total DNA was extracted using the AllPrep DNA/RNA/Protein Kit (Qiagen, Valencia, CA, USA) and quantified using the Qubit Fluorometer and dsDNA BR Assay Kit (Invitrogen, Carlsbad, CA, USA) according to the manufacturer’s instructions. mtDNA was enriched for each sample using a long-range (LR) PCR. The forward primer used was: 5′-CCGCACAAGAGTGCTACTCTCCTC-3′; the reverse primer used was: 5′-GATATTGATTTCACGGAGGATGGTG-3′ (Integrated DNA Technologies, Coralville, IA, USA)^13,55^. Each sample was amplified in a 50 μl PCR reaction that contained the following: 50 ng of total DNA, 1 μl of each 10 uM primer, 8 μl of 2.5 mM dNTPs, 0.5 μl of LA Taq DNA Polymerase, Hot-Start Version (Takara Bio USA, Inc., Mountain View, CA, USA), and 5 μl 10x buffer. Thermocycler parameters were as follows: 94 °C for 1 min, followed by 30 cycles of denaturation at 98 °C for 10 s and annealing/extension at 68 °C for 15 min, with a final extension at 72 °C for 10 min. Reactions were then kept at 4 °C. Following PCR, 5 μl (10% of the total reaction volume) was loaded into a 1% agarose gel containing 10 mg/ml ethidium bromide and the gel was run at 100 V for approximately 2 h to confirm amplification of the full-length mitochondrial genome. PCR products were purified using Agencourt AMPure XP beads (Beckman Coulter, Indianapolis, IN, USA) and were quantified using the Qubit Fluorometer and dsDNA BR Assay Kit (Invitrogen, Carlsbad, CA, USA). DNA shearing was performed in S220 Covaris microTUBEs with the following settings: Duty Factor = 5%, Peak Incident Power = 175 W, Cycles per Burst = 200, Time = 35. 200 ng of the sheared LR mitochondrial PCR product was used for library preparation using the TruSeq Nano DNA HT Library Preparation Kit (Illumina, San Diego, CA, USA). Each library was barcoded for multiplex sequencing with 96 samples per lane. NGS libraries were quantified and qualified prior to sequencing using the KAPA Library Quantification Kit (KAPA Biosystems, Wilmington, MA, USA) and Agilent Bioanalyzer DNA 7500 chips (Agilent, Santa Clara, CA, USA), respectively. Libraries were sequenced as 150-mer paired-end reads using the Illumina HiSeq 2500 at the UCI Genomics High Throughput Facility.

All accession numbers for the respective RNA-Seq datasets are provided in the Data Availability, Results section, in Supplementary Data 1 and 2, and are on GitHub (https://github.com/aomidsalar/RNA-Seq_Splice-Break2).

### Internal data preparation: bulk RNA-Seq

The internal data obtained for methods comparisons (bulk RNA-Seq and spatial transcriptomics) utilized four subject’s brain tissue obtained from the Banner Sun Health Research Institute (BSHRI) Brain and Body Donation Program. These four subjects consisted of one Parkinson’s Disease (PD) subject (144113–144114), one Alzheimer’s Disease (AD) subject (144111–144112), and two age-matched controls with no neurodegenerative disease (144105–144106 and 144107–144108). Briefly, frozen middle temporal gyrus (MTG) was microdissected into ~5 mm^3^ blocks to be compatible with the grid size used for the 10x Visium Spatial Gene Expression platform. Each block was dissected to contain a sulcus, all six cortical layers of the grey matter, and white matter. Frozen tissue was affixed to cryostat chucks with OTC and sectioned with a Microm HM525 Cryostat at −20 °C to 10 μm slices. Frozen tissue slides were adhered to the 10x Visium Spatial Gene Expression or Tissue Optimization slides following the manufacturer’s protocols. Additional sections were adhered to Selectfrost microscope slides for bulk RNA-Seq assays. All slides were frozen at −80 °C prior to library preparation.

For bulk RNA-Seq, two frozen slides from one control subject (144107/144108) were extracted for RNA using the Direct-Zol RNA mini prep kit. RNA was quantified and qualified using the Agilent Tape Station-HS RNA Screen Tape and Tape Station and had an RNA Integrity Number (RIN) of 5.6. 240 ng of RNA was used as input for each bulk RNA-Seq prep, which utilized the NEBNext Ultra II Directional RNA library prep kit. The polyA (mRNA) library preparation used the NEBNext Poly(A) mRNA Magnetic Isolation Module, and the ribo depletion library preparation used the NEBNext rRNA Depletion Kit V2 and SBP beads. Libraries were barcoded with the NEBNext Multiplex Oligos for Illumina. Library size and quality was evaluated using the Agilent Tape Station-HS DNA Screen Tape and Tape Station; libraries were quantified with the KAPA qPCR library quantification kit. Both RNA-Seq libraries were sequenced as paired-end 100-mer reads on an Illumina NovaSeq 6000 system using a NovaSeq S1 flowcell. Sequencing was performed by the USC Keck Genomics Platform (KGP).

For spatial transcriptomics, tissue optimization (i.e., permeabilization time) was tested according to the 10x Genomics protocols, with 12 min of permeabilization selected for spatial transcriptomics sequencing. Two sections of each of the four subjects that were adhered to the 10x Visium Spatial Gene Expression were processed according to the 10x Genomics Visium protocols. Hematoxylin and eosin (H&E) imaging was performed at 20X magnification and auto-stitching using a Keyence BZ-X810 Microscope to generate high-resolution TIFF files. The cDNA was amplified using 12 cycles following Cq determination steps. Library size and quality was evaluated using the Agilent Tape Station-HS DNA Screen Tape and Tape Station; libraries were quantified with the KAPA qPCR library quantification kit. Spatial transcriptomics libraries were sequenced as paired-end 150-mer reads on an Illumina NovaSeq 6000 system using a NovaSeq S4 flowcell. Sequencing was performed by the USC Keck Genomics Platform (KGP).

### Downloading files

FASTQ files available on GEO were obtained using the NCBI tool fastq-dump, with command options to split files (--split-files) and append the read ID (-I). The LIBD spatial transcriptomics dataset^26^ is available on Globus and Github; FASTQ and BAM files from this study were downloaded directly from the Globus endpoint jhpce#HumanPilot10x (http://research.libd.org/globus/jhpce_HumanPilot10x/index.html); “filtered_feature_matrix.h5” files and tissue images, which were used for clustering and image annotation, were downloaded from their Github repository (https://github.com/LieberInstitute/HumanPilot). RNA-Seq FASTQ files from cerebellum (CER), hippocampus (HIPP), and prefrontal cortex (PFC) samples were downloaded directly from the Stanley Neuropathology Consortium website after requesting access to the data portal (http://sncid.stanleyresearch.org/). GTEx samples were obtained from the AnVIL Gen3 repository and downloaded as BAM after gaining access through dbGaP; they were sorted using samtools sort^56^ (version 1.6) and converted to FASTQ using bedtools bamtofastq^57^ (version 2.25.0).

### HISAT2 alignment

In order to isolate reads that uniquely mapped to chrM and did not contain nuclear-mapped reads, we performed HISAT2^58^ alignment to the nuclear genome on the RNA-Seq samples from the Zeppillo et al. study^20^. To do this, we first downloaded the Ensembl human genome reference FASTA file version GRCh38.103^59^ and removed the mitochondrial genome (chrM) from that file. Next, we built the index files for the human genome reference using the command “hisat2-build”^58^. We then performed alignment using HISAT2^58^ default settings and the updated human genome reference file (without chrM), with the additional option “--un-conc” to store unmapped reads to separate FASTQ files. Those FASTQ files (containing reads that did not map to the nuclear genome) were then used as the input for Splice-Break2 analysis^13^.

### Splice-Break2 analysis

RNA-Seq FASTQ files were pushed through Splice-Break2 (https://github.com/brookehjelm/Splice-Break2), using either the single-end or paired-end version based on the data format of the respective study. Default command line options described were used, apart from the pre-alignment steps which were skipped (i.e., command line options: --align=yes, --ref=rCRS, fastq_keep=no, --skip_preAlign=yes). For the spatial transcriptomics datasets, alignment (BCL to BAM file) was performed with 10x Genomics Space Ranger (https://support.10xgenomics.com/spatial-gene-expression/software/overview/welcome) and BAM to FASTQ conversion was performed using the 10x Genomics bamtofastq tool (https://support.10xgenomics.com/docs/bamtofastq). The FASTQ files for Read 2 were processed through the single-end version of Splice-Break2. Single-cell samples were pooled by tissue type (via FASTQ file concatenation) prior to being run through Splice-Break2. Initial read numbers for each study were obtained from the stats file (stats.txt); all other metrics used in this analysis regarding the mtDNA read % for the “Top 30” common deletions were obtained from the “<sample > _LargeMTDeletions_DNAorRNA_Top30_NARpub.txt” files output from Splice-Break2.

### Identification of FASTQ reads spanning the 6335–13999, 7816–14807, and 8471–13449 breakpoints

We utilized the Unix command “grep” to identify reads that spanned the three deletion breakpoints described. For 6335–13999, we searched for a string containing the deletion breakpoint and ten bases upstream and downstream from that break (CTCCGTAGACCTAACCTGAC). For the 7816–14807 deletion, we searched for an 18 bp string (TCATCGACCTCCCCACCC) first, followed by strings ATCCTAGT and GAAACTTCGG, which was necessary for specificity. For the 8471–13449 deletion, we searched for a string containing the deletion breakpoint and fifteen bases upstream and downstream from that break (CAAACTACCACCTACCTCCCTCACCATTGG).

### Cortical layer imputation

We analyzed two spatial transcriptomics datasets, one from the SpatialLIBD project^26^ and the other containing internal (USC) samples (GSE226663). Spatial transcriptomics datasets were processed using the 10x Genomics Visium pipeline instructions (https://support.10xgenomics.com/spatial-gene-expression/software/overview/welcome). Scripts for spatial transcriptomics processing and analysis are available on https://github.com/aomidsalar/RNA-Seq_Splice-Break2/.

To evaluate mtDNA deletions in cortical layers using spatial transcriptomics data that did not have stereological assessment, we conducted cortical layer imputation using the SpatialLIBD results as “ground truth” since that dataset included histology. First, we performed Seurat^60^ clustering starting with “filtered_feature_matrix.h5” files on a pool of our eight USC spatial samples along with four SpatialLIBD samples (151673–151676) to generate 10 clusters, as this resulted in the most similar cortical layer architecture to the SpatialLIBD annotation. We performed further sub-clustering of cluster 3 (using the Seurat^60^ command “FindSubCluster”, to generate clusters 3_0 and 3_1), which gave us further distinction between cortical layers 2 and 3. The sample barcodes and their assigned clusters were then used to generate cluster-specific BAM files using the script “split_spatial_bam_per_cluster.py” (provided in our GitHub repository). We compared the proportion of spots that appeared in the eleven Seurat clusters to the seven “ground truth” designations (white matter and cortical layers 1–6) to assign each Seurat cluster to its corresponding imputed layer. We assigned clusters that had over 40 percent of the spots overlapping with “ground truth” annotations to that given cortical layer and omitted clusters 8 and 9 which had an overall frequency of less than 5 percent. BAM files were converted to FASTQ files using the 10x Genomics bamtofastq tool (https://support.10xgenomics.com/docs/bamtofastq); the cluster-specific FASTQ files were concatenated (when required) to make imputed cortical layer FASTQ files for each sample, which were then run through Splice-Break2.

To find spot barcodes that contained either the 6335–13999 deletion or 8471–13449 deletion, we used the Unix tool “grep” to search for the strings containing the deletion breakpoints (see Methods section “Identification of FASTQ Reads Spanning the 6335–13999, 7816–14807, and 8471–13449 Breakpoints”). As a note, the 10x Genomics bamtofastq tool will output 3 types of files for each sample: (1) a “Read 1” file, which contains the UMI and barcode; (2) a “Read 2” file, which contains the cDNA sequence; and (3) an “Index” file, which contains the sample index. Reads that contained each of these deletions (from “grep” results of Read 2) were filtered and cross-referenced with matching headers in the Read 1 FASTQ to output their corresponding barcode sequences; barcodes were then stored in separate csv files (this was done using the script “grepfilestodeletionbarcodes.sh”; https://github.com/aomidsalar/RNA-Seq_Splice-Break2), and these barcodes were used for deletion analyses. Two proportion *Z*-tests were performed by calculating the proportion of spot barcodes in each imputed cortical layer that contained a “deletion spot”; spot counting and statistics were done manually in Microsoft Excel.

#### Graphical analysis

**Fig. 1:**
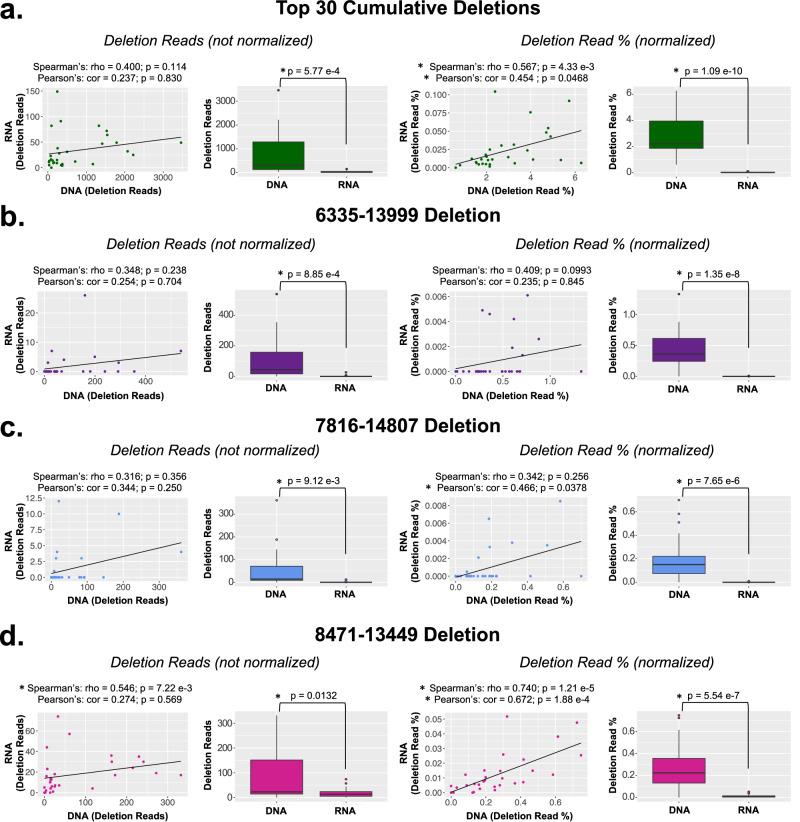
Correlations and comparisons of mtDNA deletions captured by DNA vs RNA-Seq. RNA-Seq data from 30 brain samples^20^ was processed through the Splice-Break2 pipeline and compared to results using the traditional mtDNA-enrichment and DNA sequencing approach^13,55^. All analyses included correlations and relative abundance of deletion reads detected in each sample (not normalized) and the deletion read rate for each sample (normalized). **a** The sum of the Top 30 deletions. The three most common deletions: (**b**) 6335–13999, (**c**) 7816–14807, and (**d**) 8471–13449. Spearman and Pearson’s correlations are shown. Statistical values for box plots are from Welch’s *t*-tests. All *p*-values were corrected for multiple tests using Bonferroni. All boxplots show the median as a solid black line; the first and third quartiles are captured by the bounds of the box. Boxplot whiskers are defined as the first and third quartiles ± interquartile range times 1.5, respectively, and outliers are denoted as points.

**Fig. 2:**
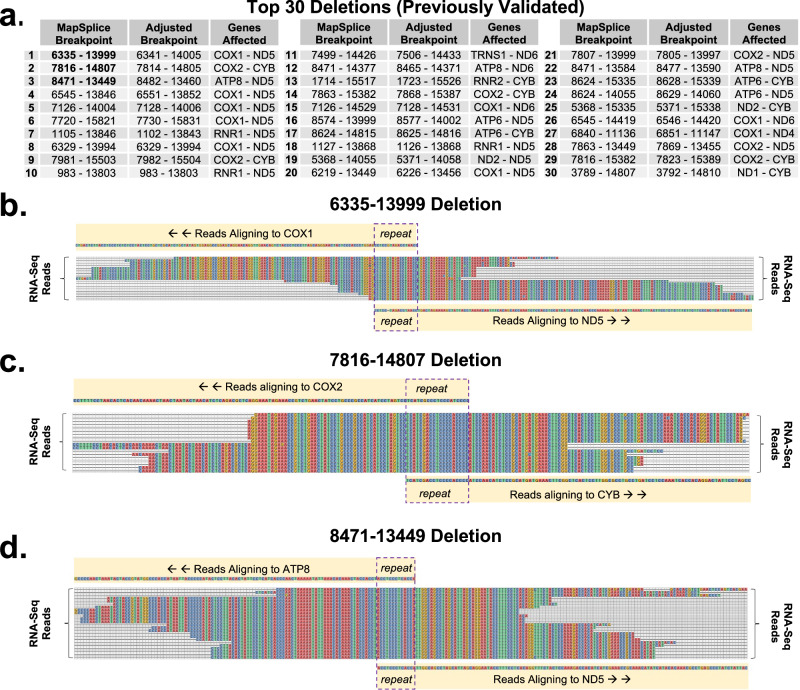
MtDNA deletions captured by RNA-Seq. **a** The Top 30 most frequent mtDNA deletions evaluated in this study and previously described^13^. Multiple sequence alignment (MSA) plot of RNA-Seq reads from a dataset of 30 brain samples^20^ containing the (**b**) 6335–13999 deletion, (**c**) 7816–14807 deletion, and (**d**) 8471–13449 deletion.

**Fig. 3:**
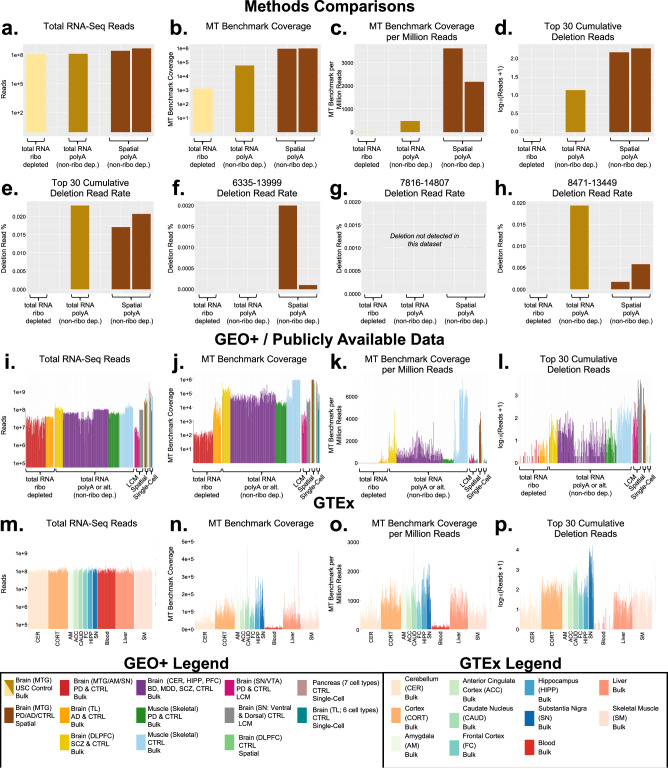
Sequencing metrics of 14 RNA-Seq datasets evaluated for mtDNA deletions. **a**–**h** USC control sample sequenced by three library preparation methods: bulk RNA-Seq with ribosomal depletion, bulk RNA-Seq without ribosomal depletion (polyA), and spatial transcriptomics (10x Visium platform). **i**–**l** All GEO+ samples (*n* = 463); (**m–p**) all GTEx samples (*n* = 1107). Definitions: Total RNA-Seq Reads = FASTQ reads prior to alignment; MT Benchmark Coverage = average mitochondrial sequencing depth measured from two 250 bp segments within the *RNR1* and *CYB* genes^13^; Deletion Read Rate = deletion reads/MT Benchmark Coverage. MTG (middle temporal gyrus); AM (amygdala); SN (substantia nigra); TL (temporal lobe); DLPFC (dorsolateral prefrontal cortex); CER (cerebellum); HIPP (hippocampus); PFC (prefrontal cortex); VTA (ventral tegmental area); LCM (laser capture microdissection); PD (Parkinson’s Disease); CTRL (control); AD (Alzheimer’s Disease); SCZ (schizophrenia); BD (bipolar disorder); MDD (major depressive disorder). Abbreviations for GTEx tissues are shown on figure.

**Fig. 4:**
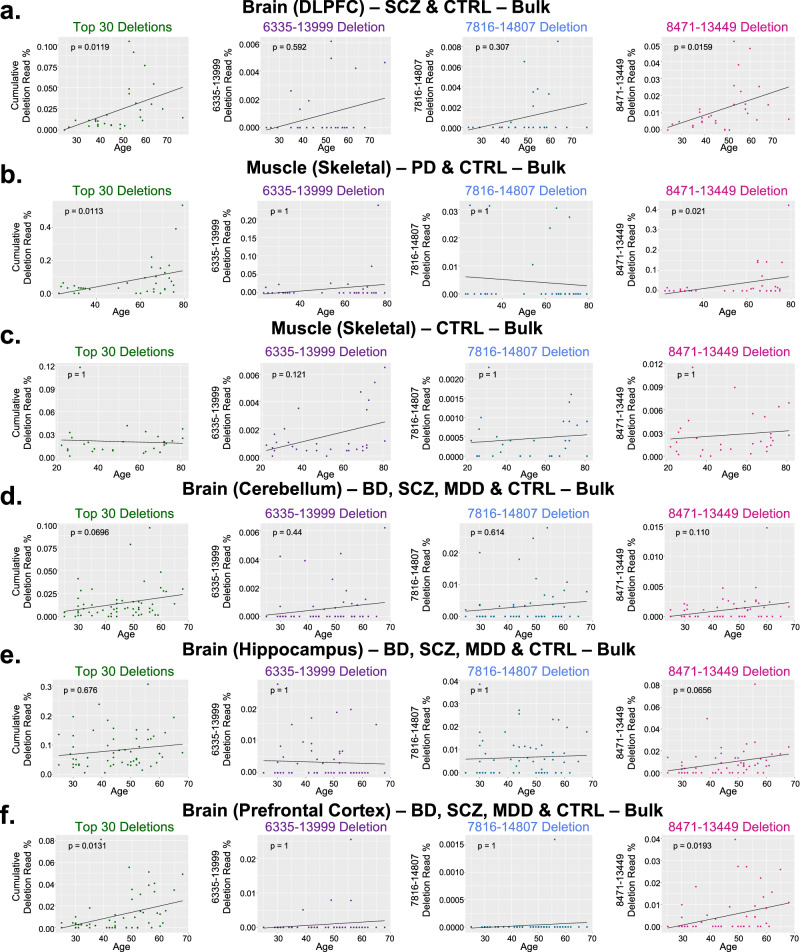
Analyses of age in GEO+ samples. Correlations between the Top 30 cumulative deletion, 6335–13999, 7816–14807, and 8471–13449 deletion read rates with age in brain^20,21^ and skeletal muscle^22,23^. **a** DLPFC samples (*n* = 30)^20^; (**b**) skeletal muscle samples (*n* = 36)^22^; (**c**) skeletal muscle samples (*n* = 30)^23^. **d**–**f** Stanley Neuropathology Consortium data^21^. **d** cerebellum samples (*n* = 58); (**e**) hippocampus samples (*n* = 58); **f** prefrontal cortex samples (*n* = 58). *P*-values shown from linear regression models for Deletion ~ Age, and include MT benchmark coverage and sex as co-variates. *P*-values in (**b**) also included diagnosis as a co-variate. All *p*-values were corrected for multiple tests using Bonferroni.

**Fig. 5:**
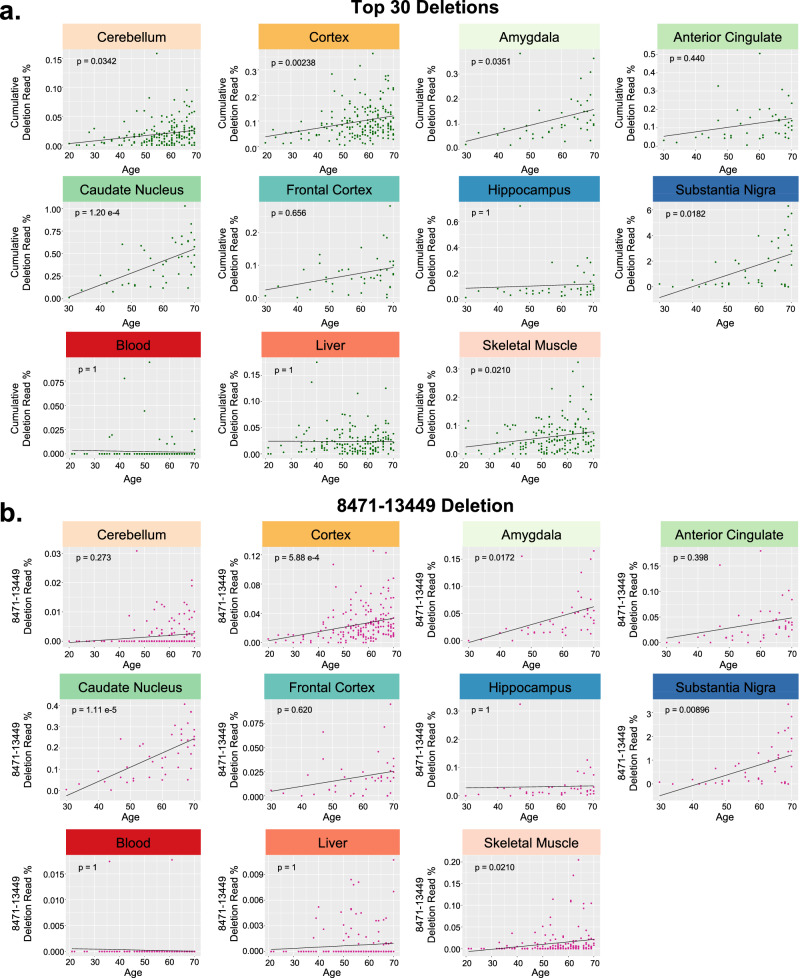
Analyses of age in GTEx samples. Correlations between the Top 30 cumulative deletion and 8471–13449 deletion read rates with age in 11 GTEx tissues^29^. **a** The cumulative deletion read rate of the “Top 30” mtDNA deletions and (**b**) the 8471–13449 deletion. Tissues are from three paired datasets: 183 paired samples of cerebellum and cortex, 41 paired samples from multiple brain regions (i.e., amygdala, anterior cingulate cortex, caudate nucleus, frontal cortex, hippocampus, and substantia nigra), and 165 paired samples from non-brain regions (i.e., blood, liver, and skeletal muscle). *P*-values shown from linear regression models for Deletion ~ Age, and include MT benchmark coverage and sex as co-variates. All *p*-values were corrected for multiple tests using Bonferroni.

**Fig. 6:**
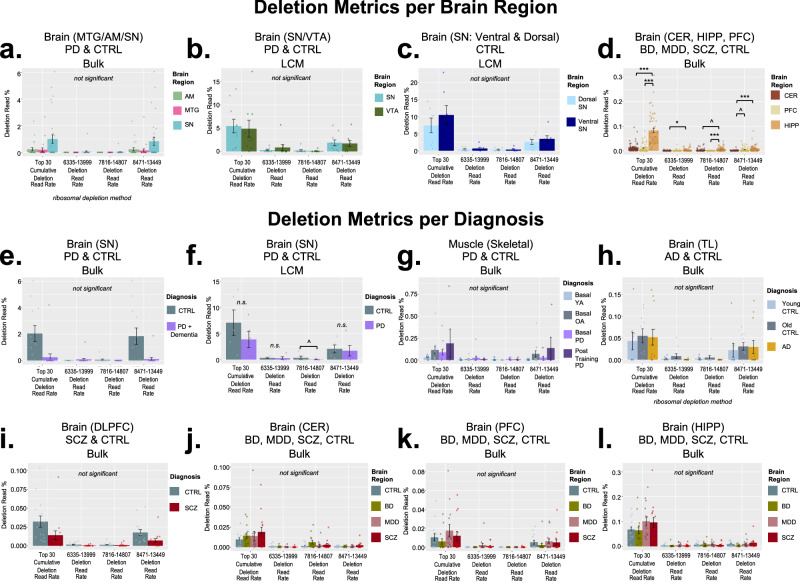
Analyses of brain region and diagnosis in GEO+ samples. **a**–**d** Comparisons between brain regions for the “Top 30” cumulative deletions, 6335–13999, 7816–14807, and 8471–13449 deletion in four studies^18,21,24,25^. **e**–**l** Comparisons of diagnosis in brain and muscle in six studies^18–22,24^. Bar graphs represent mean ± SEM. Sample size per tissue/diagnosis: (**a**) MTG (*n* = 23), AM (*n* = 23), and SN (*n* = 29)^18^; (**b**) SN (*n* = 10), VTA (*n* = 9)^24^; (**c**) dorsal SN (*n* = 7), ventral SN (*n* = 7)^25^; (**d**) CER (*n* = 58), HIPP (*n* = 58), PFC (*n* = 58)^21^; (**e**) PD+Dementia (*n* = 17), CTRL (*n* = 12)^18^; (**f**) PD (*n* = 5), CTRL (*n* = 5)^24^; (**g**) Basal YA (*n* = 12), Basal OA (*n* = 12), Basal PD (*n* = 12), Post Training PD (*n* = 5)^22^; (**h**) young CTRL (*n* = 8), old CTRL (*n* = 10), AD (*n* = 12)^19^; (**i**) SCZ (*n* = 15), CTRL (*n* = 15)^20^; (**j**) CTRL (*n* = 15), BD (*n* = 14), MDD (*n* = 15), SCZ (*n* = 14)^21^; (**k**) CTRL (*n* = 14), BD (*n* = 15), MDD (*n* = 14), SCZ (*n* = 15)^21^; (**l**) CTRL (*n* = 15), BD (*n* = 14), MDD (*n* = 15), SZ (*n* = 14))^21^. Statistical values for brain region tests are from Welch’s *t*-tests. *P*-values shown from linear regression models for Deletion ~ Diagnosis, and include MT benchmark coverage, age and sex as co-variates. All *p*-values were corrected for multiple tests using Bonferroni. MTG (middle temporal gyrus); AM (amygdala); SN (substantia nigra); VTA (ventral tegmental area); CER (cerebellum); HIPP (hippocampus); PFC (prefrontal cortex); TL (temporal lobe); DLPFC (dorsolateral prefrontal cortex); LCM (laser capture microdissection); YA (young adult); OA (older adult); PD (Parkinson’s Disease); CTRL (control); AD (Alzheimer’s Disease); SCZ (schizophrenia); BD (bipolar disorder); MDD (major depressive disorder). Symbols: (^*p* < 0.05); (*p < 0.01); (**p < 0.001); (***p < 0.0001).

**Fig. 7:**
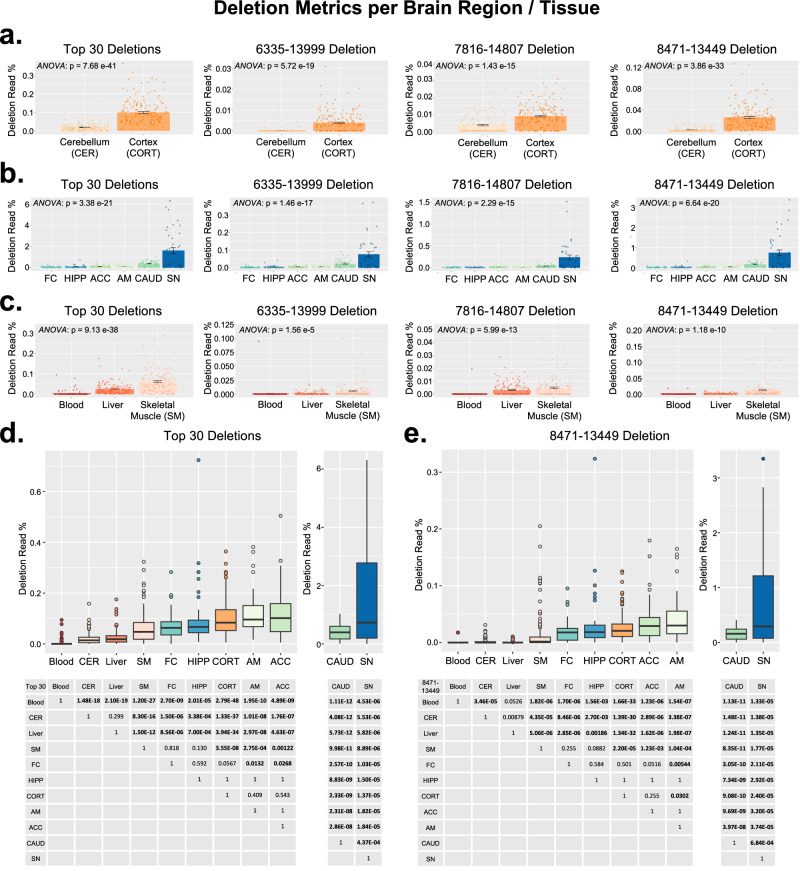
Analysis of brain region and tissue in GTEx samples. **a**–**c** Comparisons between brain regions and tissues for the “Top 30” cumulative deletions, 6335–13999, 7816–14807, and 8471–13449 deletion in paired GTEx datasets^29^. Bar graphs represent mean ± SEM. **d**, **e** Comparisons across all 11 GTEx tissues. Sample size per tissue: (**a**) paired samples from cerebellum and cortex (*n* = 183 ea.); (**b**) paired samples from amygdala, anterior cingulate cortex, caudate nucleus, frontal cortex, hippocampus, and substantia nigra (*n* = 41 ea.); (**c**) paired samples from blood, liver, and skeletal muscle (*n* = 165 ea.). **d** the “Top 30” cumulative deletions for all GTEx tissues and matrix of *p*-values for individual comparisons. **e** The 8471–13449 deletion for all GTEx tissues and matrix of *p*-values for individual comparisons. Statistical values for paired tests (**a**–**c**) are from repeated measures ANOVA. Statistical values for individual tissue comparisons (**d**, **e**) are from Welch’s *t*-tests. All *p*-values were corrected for multiple tests using Bonferroni. All boxplots show the median as a solid black line; the first and third quartiles are captured by the bounds of the box. Boxplot whiskers are defined as the first and third quartiles ± interquartile range times 1.5, respectively, and outliers are denoted as points. CER (cerebellum); CORT (cortex); FC (frontal cortex); HIPP (hippocampus); ACC (anterior cingulate cortex); AM (amygdala); CAUD (caudate nucleus); SN (substantia nigra); SM (skeletal muscle).

**Fig. 8:**
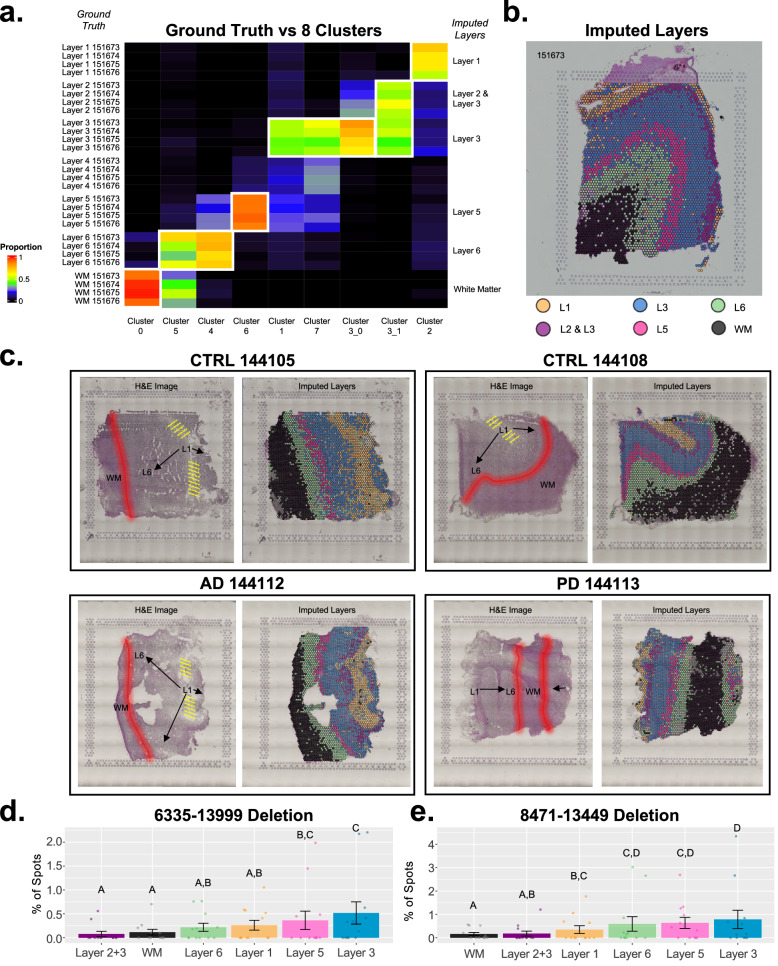
Mitochondrial deletions in spatial transcriptomics and cortical layers. **a** Heatmap showing proportion of spots corresponding to ground truth designations of four replicate SpatialLIBD^26^ samples (151673–151676) vs. Seurat clusters after integrated clustering of 12x sections (i.e., 4x SpatialLIBD DLPFC + 8x USC MTG). Clusters selected are outlined in white and imputed layers are labeled. **b** Spatial image of one SpatialLIBD sample (151673) colored by imputed cortical layers from (**a**). **c** Annotated H&E images and matching spatial images with imputed cortical from USC MTG dataset. Percentage of spots (mean ± SEM) for each imputed layer that contained (**d**) the 6335–13999 deletion or (**e**) the 8471–13449 deletion. **d**, **e** Percentages shown are from all 12x sections (4x SpatialLIBD DLPFC + 8x USC MTG). Letters above bar graphs describe significant differences between cortical layers from two-proportion *Z*-tests. Layers represented by different letters: *p* < 0.05. L1 (Layer 1); L2 & L3 (Layers 2 + 3); L3 (Layer 3); L5 (Layer 5); L6 (Layer 6); WM (White Matter).

All figures in this study (Figs. 1–8, Supplementary Fig. 1–9) were made using R (v.4.1.3); all tables were made in Microsoft Powerpoint for Mac (v.16.61). Bar graphs, scatter plots, and boxplots (Figs. 1, 3–7, 8d; Supplementary Figs. 1–7, 9) were made using the “ggplot2” package (v.3.3.5)^61^. All boxplots show the median as a solid black line; the first and third quartiles are captured by the bounds of the box. Boxplot whiskers are defined as the first and third quartiles ± interquartile range times 1.5, respectively, and outliers are denoted as points. The heatmap (Fig. 8a) was made using the “ComplexHeatmap” package (v.2.10.0)^62^. Spatial images (Fig. 8b, c; Supplementary Fig. 8) were made according to the “Secondary Analysis in R” script available on the 10x Genomics website (https://support.10xgenomics.com/spatial-gene-expression/software/pipelines/latest/rkit?src=event&lss=tradeshow&cnm=ts-2020-02-08-event-ra_g-keystone-banff-amr&cid=NULL). Multiple sequence alignment plots for the 6335–13999, 7816–14807, and 8471–13449 deletions (Fig. 2b–d) were generated using the ClustalOmega multiple sequence alignment tool on the EMBL-EBI website (https://www.ebi.ac.uk/Tools/msa/clustalo/) and the “ggmsa” R command (“ggmsa” package v.0.0.6)^63^.

#### Statistics and reproducibility

Statistical analyses were performed in both R and Microsoft Excel. Spearman’s and Pearson correlation tests were done in R using the command “cor.test”. Linear regression calculations were made using the “lm” command and ANOVA test were done using the command “aov”; *t*-tests were done using the “t.test” command in RStudio or “TTEST” formula in Microsoft Excel. Equality of variances was testing using the FTEST formula in Microsoft Excel. Two-proportion *Z*-tests were calculated manually in Microsoft Excel. All statistical tests where four deletion metrics were evaluated include multiple comparisons corrections using the Bonferroni method; this was done using the command “p.adjust” with option “method = “bonferroni” in R.

All sample sizes for the RNA-Seq studies are shown in the figure legends and in Tables 1 and 2. All replicates analyzed were from different subjects, apart from GSE140089 that included PD subjects pre- and post-exercise. All deletion metrics for GEO+ datasets are shown in Supplementary Data 2.

**Table 1:** Summary of 12 GEO + RNA-Seq datasets evaluated for mtDNA deletions

Study [Reference]	Library Preparation Kit (Method)	Age Range (yrs)	Number of Samples	Reads	Average Number of Reads [Range]	MT Benchmark Coverage [Range]	Splice-Break2 Mitochondrial (MT) Deletion Read % [Range]			
							Top 30	6335–13999	7816–14807	8471–13449
Brain (MTG/AM/SN) PD & CTRL Bulk^18^	RNAtag-Seq ribo depleted	60–95	75	84-mer SE	20.8 M [0.5–46.0 M]	105x [7–232x]	0.5120 [0–6.0481]	0.0187 [0–1.4057]	0.0088 [0–0.6589]	0.4327 [0–6.0481]
Brain (TL) AD & CTRL Bulk^19^	NEBNext Ultra Directional RNA Kit ribo depleted	42–79	30	74-mer SE	37.8 M [26.0–43.9 M]	7905x [498–46,903x]	0.0502 [0–0.1626]	0.0034 [0–0.0504]	0.0022 [0–0.0479]	0.0276 [0–0.1353]
Brain (MTG) USC Control Bulk^a^	NEBNext Ultra II Directional RNA Kit ribo depleted	82	1	101-mer PE	103.4 M	1195x	0	0	0	0
Brain (MTG) USC Control Bulk^a^	NEBNext Ultra II Directional RNA Kit non-ribo depleted	82	1	101-mer PE	119.2 M	56,577x	0.0229	0	0	0.0194
Brain (DLPFC) SCZ & CTRL Bulk^20^	Illumina TruSeq RNA Sample Prep Kit v2 non-ribo depleted	24–77	30	150-mer PE	114.5 M [65.4–234.5 M]	174,177x [46,414–568,720x]	0.0231 [0–0.1043]	0.0009 [0–0.0061]	0.00094 [0–0.0085]	0.0122 [0–0.0518]
Brain (CER, HIPP, PFC) BD, MDD, SCZ, CTRL Bulk^21^	oligo(dT) hybridization cDNA → Illumina Genomic DNA Sample Prep Kit non-ribo depleted	25–68	174	53-mer PE	65.0 M [1.7–113.8 M]	57,372x [2037–417,311x]	0.0359 [0–0.307]	0.00151 [0–0.027]	0.00327 [0–0.0385]	0.00487 [0–0.0811]
Muscle (Skeletal) PD & CTRL Bulk^22^	NuGEN Universal Plus mRNA-Seq Kit non-ribo depleted	23–79	41	50-mer PE	60.0 M [33.7–82.0 M]	18,593x [9741–32,776x]	0.0902 [0–0.844]	0.0112 [0–0.2368]	0.0045 [0–0.0315]	0.0456 [0–0.6346]
Muscle (Skeletal) CTRL Bulk^23^	NuGen Ovation v2 Preparation Kit non-ribo depleted	22–83	53	136-mer SE	107.7 M [36.7–242.4 M]	516,830x [13,765–956,995]	0.1009 [0–0.6534]	0.0030 [0–0.027]	0.0056 [0–0.1307]	0.0087 [0–0.1022]
Brain (SN/VTA) PD & CTRL LCM^24^	LCM-Seq (Smart-Seq2) non-ribo depleted	59–96	19	51-mer SE	6.3 M [1.9–10.9 M]	1908x [143–4,978x]	5.1889 [0–17.1721]	0.5623 [0–5.7112]	0.1364 [0–1.6469]	1.7982 [0–5.8016]
Brain (SN: Ventral & Dorsal) CTRL LCM^25^	LCM-Seq (SMARTer) non-ribo depleted	56–93	14	75-mer PE	96.0 M [86.5–104.9 M]	26,755x [14,201–44,940x]	8.9374 [0.599–22.892]	0.5174 [0–2.5206]	0.3247 [0–1.4603]	3.001 [0.2253–8.4713]
Brain (MTG) PD/AD/CTRL Spatial	Visium Spatial (10x Gen.) non-ribo depleted	71–83	4 subjects (8 sections)	151-mer SE	276.0 M [182.3–434.7 M]	890,023x [847,954–938,433x]	0.0827 [0.017–0.2512]	0.0185 [0.0001–0.07]	0.000925 [0–0.0051]	0.0121 [0.0008–0.0404]
Brain (DLPFC) CTRL Spatial^26^	Visium Spatial (10x Gen.) non-ribo depleted	30	1 subject (4 sections)	91-mer SE	419.3 M [233.5–822.2 M]	566,197x [390,235–892,717x]	0.0066 [0.0004–0.013]	0.000075 [0–0.0003]	0	0
Pancreas (7 cell types) CTRL Single-Cell^27^	FACS + Smart-Seq2 non-ribo depleted	1–54	7 pools (2,544 cells)	64-mer PE	1.3 B [217.1 M–3.6 B]	346,978x [44,729–754,480x]	0.00016 [0–0.0007]	0	0	0
Brain (TL; 6 cell types) CTRL Single-Cell^28^	Fluidigm + SMARTer non-ribo depleted	PN–63	6 pools (357 cells)	75-mer PE	345.5 M [70.2–836.8 M]	37,585x [6517–94,419x]	0.0032 [0–0.0191]	0	0	0

*SE* single-end, *PE* paired-end, *MTG* middle temporal gyrus, *AM* amygdala, *SN* substantia nigra, *TL* temporal lobe, *DLPFC* dorsolateral prefrontal cortex, *CER* cerebellum, *HIPP* hippocampus, *PFC* prefrontal cortex, *VTA* ventral tegmental area, *LCM* laser capture microdissection, *PD* Parkinson’s Disease, *CTRL* control, *AD* Alzheimer’s Disease, *SCZ* schizophrenia, *BD* bipolar disorder, *MDD* major depressive disorder, *M* million, *B* billion.

^a^Same study.

**Table 2:** Summary of GTEx RNA-Seq datasets evaluated for mtDNA deletions

Study [Reference]	Library Preparation Kit (Method)	Age Range (yrs)	Number of Samples	Reads	Average Number of Reads [Range]	MT Benchmark Coverage [Range]	Splice-Break2 Mitochondrial (MT) Deletion Read % [Range]			
							Top 30	6335–13999	7816–14807	8471–13449
Cerebellum Bulk^29^	Illumina TruSeq RNA Sample Prep Kit (revision A, 2010) non-ribo depleted	20–70	183	76-mer PE	98.1 M [56.5–178.9 M]	45,038x [14,095–153,168x]	0.0195 [0–0.158]	0.000222 [0–0.0125]	0.00347 [0–0.0353]	0.00179 [0–0.0308]
Cortex Bulk^29^	Illumina TruSeq RNA Sample Prep Kit (revision A, 2010) non-ribo depleted	20–70	183	76-mer PE	103.2 M [60.7–189.9 M]	95,331x [34,752–201,306x]	0.0991 [0.0058–0.364]	0.00388 [0–0.0308]	0.00886 [0–0.031]	0.0253 [0–0.125]
Amygdala Bulk^29^	Illumina TruSeq RNA Sample Prep Kit (revision A, 2010) non-ribo depleted	30–70	41	76-mer PE	101.8 M [58.8–204.6 M]	136,193x [63,196–258,796x]	0.123 [0.0167–0.382]	0.00538 [0–0.0201]	0.0103 [0–0.0393]	0.0440 [0–0.165]
Anterior Cingulate Cortex Bulk^29^	Illumina TruSeq RNA Sample Prep Kit (revision A, 2010) non-ribo depleted	30–70	41	76-mer PE	114.04 M [60.7–263.7 M]	106,615x [35,945–311,358x]	0.124 [0.0056–0.505]	0.00485 [0–0.018]	0.0152 [0–0.167]	0.0372 [0–0.180]
Caudate Nucleus Bulk^29^	Illumina TruSeq RNA Sample Prep Kit (revision A, 2010) non-ribo depleted	30–70	41	76-mer PE	109.1 M [54.2–175.1 M]	136,491x [29,799–47,6878x]	0.402 [0.0108–1.029]	0.0264 [0–0.0914]	0.0387 [0–0.156]	0.169 [0.0019–0.406]
Frontal Cortex Bulk^29^	Illumina TruSeq RNA Sample Prep Kit (revision A, 2010) non-ribo depleted	30–70	41	76-mer PE	101.3 M [63.2–149.3 M]	79,412x [12,376–135,697x]	0.0737 [0–0.283]	0.00248 [0–0.0189]	0.00797 [0–0.0231]	0.0197 [0–0.0952]
Hippocampus Bulk^29^	Illumina TruSeq RNA Sample Prep Kit (revision A, 2010) non-ribo depleted	30–70	41	76-mer PE	107.0 M [66.2–201.0 M]	133,970x [51,516–301,410x]	0.105 [0.008–0.724]	0.00484 [0–0.0416]	0.00834 [0–0.0353]	0.0331 [0–0.324]
Substantia Nigra Bulk^29^	Illumina TruSeq RNA Sample Prep Kit (revision A, 2010) non-ribo depleted	30–70	41	76-mer PE	106.2 M [69.4–163.4 M]	132,472x [34,321–278,784x]	1.618 [0–6.304]	0.0760 [0–0.372]	0.241 [0–1.512]	0.739 [0–3.348]
Blood Bulk^29^	Illumina TruSeq RNA Sample Prep Kit (revision A, 2010) non-ribo depleted	20–70	165	76-mer PE	104.3 M [61.9–221.1 M]	8,264x [1,387–27,088x]	0.00224 [0–0.0944]	0.000572 [0–0.0944]	0.000118 [0–0.0195]	0.000213 [0–0.0177]
Liver Bulk^29^	Illumina TruSeq RNA Sample Prep Kit (revision A, 2010) non-ribo depleted	20–70	165	76-mer PE	95.9 M [55.8–338.8 M]	72,406x [2,338–447,964x]	0.0241 [0–0.175]	0.000861 [0–0.0176]	0.00303 [0–0.0287]	0.000726 [0–0.0107]
Skeletal Muscle Bulk^29^	Illumina TruSeq RNA Sample Prep Kit (revision A, 2010) non-ribo depleted	20–70	165	76-mer PE	103.8 M [50.8–230.3 M]	57,885x [13,913–217,120x]	0.0610 [0–0.323]	0.00496 [0–0.0993]	0.00482 [0–0.0512]	0.0123 [0–0.205]

*SE* single-end, *PE* paired-end, *M* million.

#### Scripts and data processing details

All scripts and details on the above data processing steps are available on GitHub (https://github.com/aomidsalar/RNA-Seq_Splice-Break2). This includes the following sections: (1) “Data_Availability_and_Accession”, which includes references to datasets analyzed in this study and their corresponding accession information; (2) “Processing_FASTQ_from_GEO”, which includes scripts used to format and run single-end and paired-end FASTQ files through Splice-Break2; (3) “Grep_for_Deletions”, which includes scripts used to isolate reads containing the 6335–13999, 7816–14807, or 8471–13449 deletion reads from FASTQ files; (4) “10xSpatial_DataProcessing”, which contains scripts for generating cluster-specific BAM files and identifying barcode sequences for reads containing 6335–13999 or 8471–13449 mtDNA deletions; and (5) “Spatial_Processing_Seurat”, which contains R scripts used to perform Seurat clustering and create annotated tissue images. The GitHub repository for this paper (https://github.com/aomidsalar/RNA-Seq_Splice-Break2) and the Splice-Break2 tool repository (https://github.com/brookehjelm/Splice-Break2) both contain a document for “RNA-Seq Best Practices for Splice-Break2” to help guide users with workflow and command lines.

### Reporting summary

Further information on research design is available in the Nature Portfolio Reporting Summary linked to this article.

## Results

### Common mtDNA deletions in DNA vs RNA sequencing

We used a paired dataset of 30 postmortem brain samples that underwent both DNA and RNA-Sequencing^20^ to explore Splice-Break2’s utility in analyzing common mtDNA deletions from RNA-Seq data (Fig. 1). These samples came from DLPFC of patients with schizophrenia (SCZ) and healthy controls (CTRL). MtDNA-enriched DNA sequencing was performed as previously described^13,55^. This DNA dataset had an average of 3.47 million reads per sample, with 90.31 ± 5.2% alignment to the revised Cambridge Reference Sequence (rCRS) version of the human mitochondrial genome (NC_012920.1)^64^. This resulted in an average MT Benchmark coverage (i.e., mitochondrial depth) of 23,466x. MT benchmark coverage is a measure of the average sequencing depth of the sample, measured from two 250 bp segments within the *RNR1* and *CYB* genes^13^. RNA-Sequencing was performed as described in Zeppillo et al. ^20^. This RNA-Seq dataset had an average of 114.5 million reads per sample, with 16.17±5.9% alignment to the mitochondrial genome (NC_012920.1)^64^. Spearman and Pearson’s correlations between the Splice-Break2 results from the DNA and RNA datasets were performed for three of the “Top 30” most common mtDNA deletions, as well as the summation (cumulative read %) of all these 30 deletions together (Fig. 1). We also evaluated the summation of additional deletions of high frequency (*n* = 112), but those cumulative read %’s did not have a significant correlation between DNA and RNA (Supplementary Table 1), so we focused exclusively on the “Top 30” deletions for this investigation. The deletion read %’s for the “Top 30” deletions and the cumulative deletion read rate for every sample and this paired RNA/DNA dataset is provided in Supplementary Data 1.

The cumulative deletion read % of these “Top 30” had a significant and positive correlation between the DNA and RNA-Seq datasets after each was normalized to the amount of mitochondrial data (i.e., MT benchmark coverage) (Spearman’s rho. 0.567, *p* = 4.33e-3; Pearson’s cor. 0.454, *p* = 0.0468) (Fig. 1a). Not surprisingly, the DNA dataset had a significantly higher cumulative deletion read % than the RNA-Seq dataset (*p* = 1.09e-10), with 124-fold higher levels in the DNA (Fig. 1a). Three individual deletions were also examined for DNA/RNA correlations: the 6335–13999 deletion, which was the most frequently detected deletion in our previous study and was present in 98.9% of samples there, the 7816–14807 deletion, which was the second most frequently detected deletion and was present in 92.5% of samples there, and the 8471–13449 deletion (known as “The Common Deletion”), which was the third most frequently detected deletion in our previous study and was present in 91.4% of samples there^13^. The 6335–13999 deletion did not have statistically significant correlations (Spearman’s rho 0.409, *p* = 0.0993; Pearson’s cor. 0.235, *p* = 0.845) after multiple comparisons corrections (Fig. 1b). The DNA dataset also had a significantly higher deletion read % for this deletion than the RNA-Seq dataset (*p* = 1.35e-8), with 512-fold higher levels in the DNA (Fig. 1b). The 7816–14807 deletion did not have a significant correlation between the DNA and RNA-Seq datasets when evaluating the ranks (Spearman’s rho 0.342, *p* = 0.256); however, the correlation of read values was significant (Pearson’s cor. 0.466, *p* = 0.0378) (Fig. 1c). The DNA dataset also had a significantly higher deletion read % for this deletion than the RNA-Seq dataset (*p* = 7.65e-6), with 196-fold higher levels in the DNA (Fig. 1c). The 8471–13449 deletion had a significant and positive correlation between the DNA and RNA-Seq datasets (Spearman’s rho 0.740, *p* = 1.21e-5; Pearson’s cor. 0.672, *p* = 1.88e-4) (Fig. 1d). Again, the DNA dataset had a significantly higher deletion read % than the RNA-Seq dataset (*p* = 5.54e-7), with 22-fold higher levels in the DNA (Fig. 1d). DNA consistently had a higher deletion read % than the RNA-Seq dataset for each of the “Top 30” deletions, and a higher percentage of the “Top 30” deletions were captured in the DNA data (Supplementary Fig. 1).

The “Top 30” mtDNA deletions breakpoints from Splice-Break2, along with their adjusted positions (aligned to the first base based on the repeat sequences), and the mitochondrial genes that encompass these 5′ and 3′ breakpoints are shown (Fig. 2a). Confirmation and visualization of the three mtDNA deletions analyzed was performed using “grep” and the ggmsa R package, respectively^63^. Reads that contained the sequence CTCCGTAGACCTAACCTGAC, a 20-bp string that encompasses the 6335–13999 breakpoint, aligned as expected to the *COX1* and *ND5* genes, with retainment of 1 copy of the repeat sequence (Fig. 2b). Reads that contained the sequence TCATCGACCTCCCCACCC an 18-bp string that encompasses the 7816–14807 breakpoint (and that also contained the sequences ATCCTAGT and GAAACTTCGG), aligned as expected to the *COX1* and *CYB* genes, with retainment of 1 copy of the repeat sequence (Fig. 2c). Reads that contained the sequence CAAACTACCACCTACCTCCCTCACCATTGG, a 30-bp string that encompasses the 8471–13449 breakpoint, aligned as expected to the *ATP8* and *ND5* genes, with retainment of 1 copy of the repeat sequence (Fig. 2d).

To determine if nuclear-mitochondrial DNA segments (NUMTs) were contributing to our detection of common mtDNA deletions, and to assess if it was necessary to process RNA-Seq data with these sequences removed, we compared the mtDNA deletion results from all reads to only those that uniquely mapped to chrM (i.e., after HiSat2^58^ alignment to the nuclear genome and downstream processing of unmapped reads) (Supplementary Fig. 2). The correlations between the mtDNA deletion reads detected (before normalization) was very high for both the “Top 30” sum and the three individual deletions evaluated when comparing all reads to only uniquely mapped reads (Spearman’s rho range: 0.81 to 0.999, *p*-value range: 2.40e-7 to 2.72e-42; Pearson’s cor range: 0.957–0.998, *p*-value range: 2.06e-20 to 5.10e-39). There was no significant difference in either the number of deletion reads detected or the deletion read % (after normalization) for any deletion metric (Supplementary Fig. 2). Additional analysis of the uniquely mapped reads from RNA-Seq data compared to the DNA dataset demonstrated that utilizing uniquely mapped reads did not significantly improve the correlations between RNA and DNA (Supplementary Fig. 3); thus, we concluded removal of nuclear mapped reads was not necessary and we processed all RNA-Seq reads through the Splice-Break2 pipeline for the remainder of this study to streamline our workflow. However, both approaches (i.e., using all reads or only chrM uniquely mapped reads) are described in our “RNA-Seq Best Practices for Splice-Break2” document on GitHub.

### Total reads, mitochondrial data and common mtDNA deletions across 14 RNA-Seq studies

We processed 14 human RNA-Seq studies^18–29^ through the Splice-Break2 pipeline and evaluated the effects of library preparation method and sequencing depth (i.e., total read numbers and MT benchmark coverage) on our ability to capture common mtDNA deletions (Fig. 3). Our analysis included 12 previously published studies and 2 newly presented here. The 12 previously published studies we included are as follows (see Tables 1 and 2 for additional details): (1) Simchovitz et al. (GSE114517) study of ribosomal-depleted RNA-Seq libraries isolated from substantia nigra (SN), amygdala (AM), and middle temporal gyrus (MTG) brain tissue of patients with PD+Dementia and CTRL^18^; (2) Nativio et al. (GSE159699) study of ribosomal-depleted RNA-Seq libraries isolated from lateral temporal lobe (TL) tissue of patients with AD and CTRL^19^; (3) Zeppillo et al. study (GSE224683) of polyA (non-ribosomal depleted) total RNA libraries isolated from DLPFC brain tissue of patients with SCZ and CTRL^20^; (4) Kim et al. study (i.e., The Stanley Neuropathology Consortium) of polyA (non-ribosomal depleted) total RNA libraries isolated from cerebellum (CER), hippocampus (HIPP), and prefrontal cortex (PFC) of patients with bipolar disorder (BD), SCZ, major depressive disorder (MDD) and CTRL^21,65^; (5) Lavin et al. (GSE140089) study of polyA (non-ribosomal depleted) total RNA libraries isolated from skeletal muscle (SM) of patients with PD and CTRL^22^; (6) Tumasian et al. (GSE164471) study of polyA (non-ribosomal depleted) total RNA libraries isolated from SM of CTRL^23^; (7) Aguila et al. (GSE114918) study of laser-capture microdissection (LCM) SN and ventral tegmental area (VTA) neurons isolated from patients with PD and CTRL^24^; (8) Monzón-Sandoval et al. (GSE166024) study of LCM ventral and dorsal SN neurons isolated from CTRL subjects^25^; (9) Maynard et al. spatial transcriptomics sequencing of DLPFC brain tissue isolated from CTRL subjects^26^; (10) Enge et al. (GSE81547) analysis of 2544 single cells (alpha, acinar, beta, delta, ductal, mesenchymal, and unsure cell types) isolated from pancreas tissue of CTRL subjects^27^; (11) Darmanis et al. (GSE67835) analysis of 357 single cells (neurons, fetal quiescent, astrocytes, endothelial, oligodendrocyte precursor cells, and microglia cell types) isolated from fresh brain tissue^28^; and (12) Lonsdale et al. (i.e., GTEx) study, evaluating multiple brain regions, skeletal muscle, liver and blood^29^. Single-cell samples were pooled by cell type prior to Splice-Break2 analysis, resulting in 7 pools of pancreas cell types and 6 pools of brain cell types (Table 1). The two newly presented studies include one bulk sequencing dataset of postmortem MTG brain tissue with RNA libraries prepared both with (*n* = 1) and without (*n* = 1) ribosomal depletion, as well as one spatial transcriptomics dataset of MTG brain tissue from patients with PD (*n* = 2), AD (*n* = 2), and CTRL (*n* = 4) (GSE226663). One of our internal control samples was sequenced using all three preparation methods; this helped us compare the effect of library preparation method on our ability to capture mtDNA deletions without additional effects of tissue type or sequencing site. In total, we examined 1570 samples ranging in age from prenatal to 96 years across the 14 datasets.

We processed all 1570 samples (Fig. 3a–p), including the one internal (MTG) sample that had been processed three ways (Fig. 3a–h), through the Splice-Break2 pipeline and compared the following key metrics to determine which variables influenced common mtDNA deletion levels: total RNA-Seq reads, mitochondrial (MT) benchmark coverage, MT benchmark coverage per million reads, and the “Top 30” cumulative deletion read numbers. Total RNA-Seq reads are the initial reads from each sample prior to alignment. MT benchmark coverage is a measure of the average sequencing depth of the sample, measured from two 250 bp segments within the *RNR1* and *CYB* genes^13^. The “Top 30” cumulative deletion read amount is the sum of the “Top 30” reads. The deletion read rates for the internal samples processed three ways are also shown (Fig. 3e–h), which are calculated by normalizing the number of reads for each mtDNA deletion to the MT benchmark coverage, and is the metric used to evaluate the effect of age, tissue, and diagnoses (see Figs. 4–7). The deletion read %’s for the “Top 30” deletions and the cumulative deletion read rate for every sample and all RNA-Seq studies is provided in Supplementary Data 2 for the GEO+ datasets; individual-level data is not shown for the GTEx dataset because of dbGaP restrictions.

We observed that samples that used a ribosomal depletion library preparation method (but had similar abundance of sequencing reads prior to alignment) had less MT benchmark coverage than polyA (non-ribosomal depleted) samples (Fig. 3a–c, i–k). Due to a reduced amount of mitochondrial data in the samples subjected to ribosomal depletion, we observed little to no common mtDNA deletions in these samples; conversely, for samples prepared without ribosomal depletion (i.e., bulk polyA or other), we consistently observed these common mtDNA deletions and more mitochondrial data overall (Fig. 3). Samples prepared by LCM showed the highest number of deletion reads (Fig. 3l); however, it is important to note that these LCM samples were all derived from aged human brain regions with high dopaminergic neuron innervation, the SN and VTA, so these high values may not solely reflect the sample preparation method. Evaluations of our internal control samples prepped by three methods further confirmed our observation of higher abundance of mitochondrial reads and deletions in polyA (non-ribosomal depleted) preparations and showed more MT data was captured by spatial transcriptomics than by bulk RNA-Seq (Fig. 3b–h). This is in alignment with previous observations that this spatial transcriptomics method (i.e., 10x Genomics Visium) may capture more “off-target” and/or non-polyadenylated reads compared to other methods^26,66^. Single-cell RNA sequencing (scRNA-Seq) samples did not contain the three mtDNA deletions followed in this study, so these samples were not evaluated further (Table 1, Fig. 3l). Taken together, we found that bulk sequencing with polyA (non-ribosomal depletion), spatial transcriptomics, and LCM methods were amenable to mtDNA deletion investigations, while scRNA-Seq and ribosomal depletion methods were generally inutile due to insufficient mitochondrial data.

### Common mtDNA deletions and the effects of age and sex

MtDNA mutations and deletions have been positively associated with aging in various tissues such as the brain, skeletal muscle, and heart^4,5,43,67–70^. One of the most investigated large mtDNA deletions is the 8471–13449 “common deletion” (also referred to as the 4977 bp deletion), which was found to increase with age in the heart and brain of healthy adult patients^43,69,70^. In contrast with the nuclear genome, the mitochondrial genome is particularly vulnerable to DNA damage because it is located in close proximity to damaging free radicals and reactive oxygen species generated through the OXPHOS pathway, does not have the protection of histones, and has less efficient DNA repair machinery^71–73^. Over time, these factors can lead to the accumulation of mtDNA deletions within cells^70,71,73^.

Among the GEO+ datasets, we chose four studies for age analysis because of the wide age distributions of their samples^20–23^. In the DLPFC study^20^, we observed significant positive correlations between age and the “Top 30” cumulative deletion read rate (*p* = 0.0119) and the 8471–13449 “common deletion” (*p* = 0.0159) (Fig. 4a). In the SM study from PD and CTRL samples^22^, we similarly observed significant positive correlations between age and the “Top 30” cumulative deletion read rate (*p* = 0.0113) as well the 8471–13449 “common deletion” (*p* = 0.021; Fig. 4b). In the SM study of CTRL samples^23^, we did not observe a significant correlation between age and any of the four mtDNA deletion metrics evaluated (Fig. 4c); only ~1/2 of the samples were included in the aging analysis because we observed a significant “batch effect” in this study (Supplementary Fig. 4). In the Stanley Neuropathology Consortium dataset^21^, which contained CER, HIPP, and PFC, we observed significant positive correlations between age and the “Top 30” cumulative deletion read rate (*p* = 0.0131) and the 8471–13449 “common deletion” (*p* = 0.0193) in the PFC (Fig. 4f); these deletion metrics were also observed to increase with age in the CER and HIPP, but the correlations with age were not significant after multiple-comparisons corrections (Fig. 4d, e). We did not observe a significant correlation with age for the 6335–13999 or 7816–14807 deletion in any of these datasets (Fig. 4). In addition, none of these datasets exhibited significant differences between biological sexes for any of the four deletion read rate metrics (Supplementary Table 2).

Age analysis for the GTEx RNA-Seq data was also performed on a subset of available samples, including 183 paired samples of cerebellum (CER) and cortex (CORT), 41 paired samples from multiple brain regions (i.e., amygdala (AM), anterior cingulate cortex (ACC), caudate nucleus (CAUD), frontal cortex (FC), hippocampus (HIPP), and substantia nigra (SN)), and 165 paired samples from non-brain regions (i.e., blood, liver and skeletal muscle (SM)) (Fig. 5 and Supplementary Fig. 5). In the paired CER and CORT dataset, we observed significant positive correlations between age and the “Top 30” cumulative deletion read rate (*p* = 0.00238) and the 8471–13449 “common deletion” (*p* = 5.88e-4) in the CORT, and also a significant positive correlation between age and the “Top 30” cumulative deletion read rate (*p* = 0.0342) in the CER (Fig. 5). The other common deletions evaluated were not significant for these brain regions after multiple comparisons corrections (Fig. 5 and Supplementary Fig. 5). In the paired analysis of 6 brain regions, we observed significant positive correlations between age and the mtDNA deletion metrics for the AM, CAUD and SN; we did not observe significant correlations with age in the ACC, FC or HIPP after multiple comparisons corrections (Fig. 5 and Supplementary Fig. 5). Significant age correlations in the AM included the 8471–13449 deletion (*p* = 0.0172) and the “Top 30” cumulative deletion read rate (*p* = 0.0351). Significant age correlations in the CAUD included the 6335–13999 deletion (*p* = 1.94e-3), the 7816–14807 deletion (*p* = 0.0358), the 8471–13449 deletion (*p* = 1.11e-5), and the “Top 30” cumulative deletion read rate (*p* = 1.20e-4). Significant age correlations in the SN included the 8471–13449 deletion (*p* = 0.00896), and the “Top 30” cumulative deletion read rate (*p* = 0.0182). Lastly, in the paired analysis of blood, liver and SM, we only observed significant and positive correlations between age and the mtDNA deletion metrics for SM, specifically for the 8471–13449 deletion (*p* = 0.0210), and the “Top 30” cumulative deletion read rate (*p* = 0.0210) (Fig. 5). Overall, we were able to recapitulate previously published findings that common mtDNA deletions increase with age in the brain and muscle, and we provide evidence from paired datasets that different brain regions and tissues have variable susceptibility to this increase, some of which, to our knowledge, is illustrated for the first time.

### Common mtDNA deletions across brain regions, tissues, and the effect of diagnosis

Evaluation of the MTG, AM, and SN dataset^18^ of PD and CTRL subjects revealed no significant differences in “Top 30” cumulative deletion read % in the SN compared to the MTG or AM for any of the deletion metrics, after multiple comparisons corrections (Fig. 6a). However, the SN did display higher levels and it should be emphasized that this study used a ribosomal depletion method for library preparation, so it is perhaps not surprising we were unable to detect a significant difference since so little mitochondrial data is captured with that RNA-Seq method. The SN and VTA LCM dataset^24^ from PD and CTRL samples (Fig. 6b) and the dorsal and ventral SN LCM dataset^25^ of CTRL subjects (Fig. 6c) showed no significant differences in any of the four mtDNA deletion metrics we evaluated. The Stanley Neuropathology Consortium dataset^21^ of CER, PFC, and HIPP tissue showed significantly higher deletion read %’s in the HIPP compared to CER and PFC for the 7816–14807 deletion (*p* ≤ 0.01324) and the “Top 30” cumulative deletion read rate (*p* ≤ 3.12e-12; Fig. 6d). The HIPP had significantly higher deletion read %’s than the CER for the 6335–13999 deletion (*p* = 0.00636) and the 8471–13449 deletion (*p* = 2.92e-5) (Fig. 6d). The 8471–13449 deletion were also significantly higher in the PFC than in the CER (*p* = 0.0261) (Fig. 6d).

In general, we did not detect significant differences based on diagnosis in any of the GEO+ datasets we evaluated. The one exception to this was the SN and VTA LCM dataset^24^ from PD and CTRL samples had significantly higher levels of the 7816–14807 deletion in the CTRL subjects in both SN (*p* = 0.0109) and VTA (*p* = 0.0064) (Fig.6f and Supplementary Fig. 6). It should be noted that all of these studies had a small sample size per diagnostic group (range: *n* = 5–17).

Analysis of GTEx included tissue comparisons of the three paired datasets used in our aging analysis (Fig. 5). In paired CER and CORT, the CORT had significantly higher amounts of all four deletion metrics (6335–13999 deletion (*p* = 5.72e-19); 7816–14807 deletion (*p* = 1.43e-15); 8471–13449 deletion (*p* = 3.86e-33); and the “Top 30” cumulative deletion read rate (*p* = 7.68e-41) (Fig. 7a). Across the six paired brain regions, deletion levels were also significantly different (6335–13999 deletion (*p* = 1.46e-17); 7816–14807 deletion (*p* = 2.29e-15); 8471–13449 deletion (*p* = 6.64e-20); and the “Top 30” cumulative deletion read rate (*p* = 3.38e-21) (Fig. 7b). Similarly, in paired blood, liver and SM, all four deletion metrics were significantly different (6335–13999 deletion (*p* = 1.56e-5); 7816–14807 deletion (*p* = 5.99e-13); 8471–13449 deletion (*p* = 1.18e-10); and the “Top 30” cumulative deletion read rate (*p* = 9.13e-38) (Fig. 7c). Cross comparisons of all GTEx tissues revealed the following results: (1) blood had the lowest deletion read % and was significantly lower than all other tissues; (2) CER had significantly lower deletion levels than all other brain regions; (3) liver had significantly lower deletion levels than SM and cortical brain regions; (4) SM had significantly lower deletion levels than some (but not all) brain regions; (5) brain regions with dopaminergic neurons (i.e., the CAUD and SN) had remarkable enrichment of common mtDNA deletions, with significantly higher levels than all other tissues. The SN had by far the highest mtDNA deletion levels and was likewise significantly higher than all tissues including the CAUD. All tissue comparisons and *p*-values are shown in Fig. 7 and Supplementary Fig. 7.

### Common mtDNA deletions across imputed cortical layers in spatial transcriptomics data

To investigate the common mtDNA deletion metrics across (imputed) cortical layers, we used 12 spatial transcriptomics samples from two separate human brain datasets. The two spatial transcriptomics datasets include the Maynard et al. “SpatialLIBD” dataset^26^, from which we included 4x section replicates of DLPFC tissue from a single healthy control subject (sample numbers 151673–151676), and a new internal dataset of 8x spatial transcriptomics samples of MTG tissue taken from duplicate sections of 4 patients (diagnosed as AD, PD, or CTRL). Both datasets were prepared according to 10x Genomics’s Visium spatial transcriptomics library preparation and sequencing protocols. The SpatialLIBD samples were included to assist in cortical layer imputation since they contained “ground truth” measures of cortical layer geography based on stereology/imaging analysis^26^. We performed Seurat^60^ clustering on all 12 spatial samples from both studies and imputed cortical layers based on the Seurat clusters that had the highest overlap with the SpatialLIBD ground truth annotations; this resulted in the following imputed cortical layers: “White Matter”, “Layer 1”, “Layer 2 + 3”, “Layer 3”, “Layer 5”, and “Layer 6” (Fig. 8a–c).

To determine the proportion of spots in each cortical layer that contained the 6335–13999 and/or 8471–13449 mtDNA deletion, we needed the spot barcode data (which is not output as part of the Splice-Break2 process). As such, we used “grep” to identify reads that spanned these breakpoints, as described previously, and then determined which cortical layer these reads mapped to using the spot barcode. The percentage of spots that contained the 6335–13999 deletion (Fig. 8d) or the 8471–13449 deletion (Fig. 8e) for all 12 samples was analyzed for each imputed cortical layer. We observed that the 6335–13999 deletion had a significantly higher percentage of spots (two-proportion *Z*-test) in imputed cortical layers 3 and 5 compared to the white matter (*p* ≤ 0.0066; Fig. 8d). The 8471–13449 “common deletion” also had significantly higher geographical representation in cortical layers 3 and 5 compared to white matter (*p* ≤ 3.30e-5; Fig. 8e). These spots containing deletions were also annotated on tissue images for visualization (Supplementary Fig. 8). Further, we observed similar trends in the percentage of spots with the 6335–13999 or 8471–13449 deletion when analyzing just the USC internal samples (Supplementary Fig. 9). These 8x spatial samples were split according to their imputed cortical layer (i.e., using “cluster BAMS” from Seurat^60^), and each imputed layer was run through Splice-Break2; we found a significant difference between white and grey matter for the 8471–13449 “common deletion” (*p* = 0.0393; Supplementary Fig. 9). Taken together, these results suggest cortical layers have different levels of common mtDNA deletions in the aged human brain and are most abundant in layers 3 and 5 where there is an enrichment of pyramidal neurons^26,74,75^.

## Discussion

The aim of this study was to determine whether RNA-Seq data can be used to evaluate common mtDNA deletion levels in somatic tissues using the bioinformatics tool Splice-Break2. Initial analyses were done on a paired dataset where there was DNA and RNA-Seq data available; we observed robust differences in DNA and RNA deletion read %, with the DNA data containing *at least* 22-fold higher deletion read rates. We hypothesize that the decreased rate of these common deletions in RNA-Seq data may be due to transcript abundance or stability- that mitochondrial genomes with large deletions may transcribe polycistronic transcripts less efficiently than wild-type molecules, or that RNA transcripts containing deletion breakpoints may be less stable, or both. It is worth mentioning that polyadenylation of mitochondrial transcripts serves a different role than it does for nuclear-encoded genes where 3′ polyadenylation helps stabilize mRNA molecules and promotes protein translation; this also occurs for human mitochondrial-encoded genes, but transcripts can also be polyadenylated at many intragenic positions and are subject to polyadenylation-dependent RNA degradation mechanisms that are conserved features of bacteria (the evolutionary origin of mitochondria)^76–78^. In addition, the deletion read %’s in the DNA data are likely increased due to the long-range PCR amplification used for mitochondrial enrichment and the smaller size of the deleted genomes.

The mtDNA deletion metrics that were examined in this study were the cumulative read % of the “Top 30” most frequent deletions that we identified and Sanger-validated in our previous study, and the individual read % of three specific mtDNA deletions: 6335–13999 (adjusted position 6341–14005), 7816–14807 (adjusted position 7814–14805), and 8471–13449 (the “common deletion”; adjusted position 8482–13460). The cumulative read % of the “Top 30” deletions and the 8471–13449 deletion had statistically significant positive correlations between the DNA and RNA-Seq paired dataset examined, with the “common deletion” having the strongest correlations between methods. Future studies may include additional paired datasets (DNA, RNA-Seq, and other genomics methods) and analyses of less common mitochondrial deletions. Analysis of patients with large clonal deletions will also be of interest. The Splice-Break2 pipeline does output additional breakpoints besides the “Top 30” most common deletions, but those metrics should be used with caution for RNA-Seq data and may require further validation (such as qPCR and/or Sanger sequencing).

All Splice-Break2 data is reported as “deletion read %”, which is the number of deletion reads detected divided by the MT Benchmark coverage; however, this should not be interpreted as an absolute estimation of heteroplasmy rate (for DNA or RNA-Seq) because of inherent biases of these NGS methods. In our original methods article, we did observe a significant positive correlation between the Splice-Break2 deletion read % and qPCR quantification of the 8471–13449 “common deletion”, and here we show the deletion read %’s can correlate between DNA and RNA-Seq datasets- thus, we conclude that although absolute heteroplasmy rates cannot be inferred, the fold differences between samples is retained and allows us to use deletion read % for statistical analyses of age, tissue type and diagnoses. Future studies that pair mitochondrial/metabolic assays with NGS measurements will be important to understand the physiological relevance of these deletions and at what threshold deletion read %’s impact cellular function.

Splice-Break2 was used to examine various library preparation methods, such as bulk RNA-Seq, LCM RNA-Seq, spatial transcriptomics, and scRNA-Sequencing. We observed that bulk sequencing without ribosomal depletion allowed for more consistent capturing of mtDNA transcripts and deletions compared to bulk sequencing with ribosomal depletion. Additionally, the scRNA-Seq studies we evaluated had minimal to no mtDNA deletion capture despite containing comparable amounts of mtDNA transcripts (MT Benchmark coverage) compared to the other studies evaluated. Analysis of additional scRNA-Seq datasets and methods, including LCM, along with other non-RNA single-cell genomics methods (such as scATAC-Seq), is warranted in the future. The LCM RNA-Seq samples we evaluated contained the highest deletion read rates; however, it is important to note that these samples originated from aged brain tissue from regions innervated with dopaminergic neurons (SN and VTA), which have a demonstrated significant increase in mtDNA deletion burden^79,80^. Therefore, we cannot determine from these LCM datasets if the increased mtDNA deletion levels are strictly due to brain region, library preparation method, or are a combination of both effects. Overall, these data indicate that the most valuable RNA-Seq wet lab protocols for mtDNA deletion detection include bulk sequencing without ribosomal depletion (e.g., polyA), LCM RNA-Seq, and spatial transcriptomics.

Our analysis of aging, differences among brain regions, and diagnostic effects revealed trends that were consistent with the effects of mitochondrial dysfunction reported in current literature^2–5,9,79,80^. It has been reported in several studies that mtDNA deletions accumulate in some tissues as they age, and studies on the “hallmark” mtDNA deletion disorders (e.g., KSS, Pearson’s Syndrome) have proved these deletions can have functional (and even lethal) consequences if at a high enough threshold to affect cellular function^36,37^. The age-dependent increase in mtDNA deletions has been observed in various somatic tissues including the brain; in the substantia nigra, high mtDNA deletions have been linked to age-related and disease-related (i.e., PD) neuronal loss^43,67,79,80^. We observed statistically significant increases in the mtDNA deletion metrics due to age in brain and/or muscle datasets. Our most robust evaluation of aging was performed using paired RNA-Seq data from GTEx. Overall, we were able to recapitulate previously published findings that common mtDNA deletions increase with age in the brain and muscle, and show that different brain regions and tissues have variable susceptibility to this age-dependent increase.

We were able to observe some interesting differences in tissues and brain regions in the paired GTEx dataset, where common mitochondrial deletions were lowest in blood, lower in liver than skeletal muscle, higher in cortex than in cerebellum, and high (but variable) across cortical brain regions. In both GEO+ and GTEx datasets, we observed increased mtDNA deletions in brain regions containing dopaminergic neurons (SN, VTA and caudate nucleus); however, we did not detect an increase in PD. This is consistent with previous reports that have found an increase in SN tissue but no significant increase in PD specifically^79,81–83^. It would be interesting to investigate a larger cohort of patient samples taken at various stages of PD and/or compare these results neuropathological measures of dopaminergic cell death (e.g., SN depigmentation scores), as this data suggests the age effects in PD SN may not be linear. Measures of cell type abundance (e.g., histology, in situ gene expression, cell counts from flow cytometry or CBC tests, or single-cell RNA-Seq data) from paired tissue (preferably the same dissection/sample) maybe also improve analyses of age and diagnosis if that data can be obtained and used as a co-variate.

Our common mtDNA deletion analysis of spatial transcriptomics data, specifically across (imputed) cortical layers of the DLPFC and MTG, revealed increased deletion burden in grey matter compared to white matter, which is not surprising given its high metabolic activity^74,84,85^. Cortical layers 3 and 5 consistently contained the highest percentage of “spot barcodes” with mtDNA deletions. This supports the hypothesis that brain regions and cortical layers are differentially susceptible to mtDNA damage^69,79,80,86^. Future studies investigating the effect of mtDNA damage in neurodevelopment, psychiatric disorders or neurodegenerative diseases may want to focus on spatial transcriptomic approaches so that analyses can focus on specific cortical layers and increase the signal-to-noise ratio for these tests.

In summary, this robust analysis of multiple, highly used RNA-Seq methods demonstrated the utility of detecting mtDNA deletions of high frequency through our bioinformatics pipeline. The Splice-Break2 tool was effective in quantifying common mtDNA deletions in polyA (non-ribosomal depleted) bulk sequencing, LCM, and spatial transcriptomic datasets, and these datasets may also be amenable to other bioinformatics approaches of mtDNA deletion quantification. Of note, the current version of Splice-Break2 is only compatible with human data, as the alignment and annotations utilize the rCRS^64^, our catalogue of human mtDNA deletion breakpoints^13^, and MitoMap^87^, respectively. With the wide breadth of publicly available and restricted human RNA-Seq datasets that encompass a variety of tissues, diseases, and experimental/environmental conditions, the ability to incorporate mtDNA deletion metrics into these investigations may provide information about metabolic effects in tissues and their contributions to disease phenotypes and aging.

### Supplementary information

Supplementary Information

Supplementary Data 1

Supplementary Data 2

Supplementary Data 3

Reporting Summary

## Data Availability

Our analysis included 12 previously published studies and 2 newly presented here. The 12 previously published studies we included in this paper can be accessed from the following: (1) Simchovitz et al.^18^ study is deposited on GEO with accession code “GSE114517”; (2) Nativio et al.^19^ study is deposited on GEO with accession code “GSE159699”; (3) Zeppillo et al.^20^ study is deposited on GEO with accession code “GSE224683”; (4) Kim et al. study^21,65^ (i.e., The Stanley Neuropathology Consortium) is available on their website (http://sncid.stanleyresearch.org); 5) Lavin et al. study^22^ is deposited on GEO with accession code “GSE140089”; (6) Tumasian et al. study^23^ is deposited on GEO with accession code “GSE164471”; (7) Aguila et al. study^24^ is deposited on GEO with accession code “GSE114918”; (8) Monzón-Sandoval et al. study^25^ is deposited on GEO with accession code “GSE166024”; (9) Maynard et al. study^26^ is available on their GitHub page (https://github.com/LieberInstitute/HumanPilot) and Globus endpoint “jhpce#HumanPilot10x” (http://research.libd.org/globus/jhpce_HumanPilot10x/index.html); (10) Enge et al. study^27^ is deposited on GEO with accession code “GSE81547”; (11) Darmanis et al. study^28^ is deposited on GEO with accession code “GSE67835”; and (12) Lonsdale et al. (i.e., GTEx) study^29^ is available through their portal (https://gtexportal.org/home/)^29^. All RNA-Seq samples newly presented in this study have been deposited on GEO with primary accession code “GSE226663”. Source data underlying Fig. 1 and Fig. 3a–h can be found in Supplementary Data 1. Deletion metrics for all GEO+ studies (used for Figs. 3, 4 and 6) can be found in Supplementary Data 2. Source data underlying Fig. 8d can be found in Supplementary Data 3. Raw data is not provided for the GTEx datasets due to restricted access.

## Code availability

All scripts and details on processing steps are available (https://github.com/aomidsalar/RNA-Seq_Splice-Break2; 10.5281/zenodo.10499375)^88^. This repository and the Splice-Break2 tool repository (https://github.com/brookehjelm/Splice-Break2; 10.5281/zenodo.10499097)^89^ both contain a document for ‘RNA-Seq Best Practices for Splice-Break2’ to help guide users with workflow and command lines.

## References

[1] Holt IJ, Harding AE, Morgan-Hughes JA (1988). Deletions of muscle mitochondrial DNA in patients with mitochondrial myopathies. Nature.

[2] Wallace DC (1988). Mitochondrial DNA mutation associated with Leber’s hereditary optic neuropathy. Science.

[3] Wallace DC (1995). Mitochondrial DNA mutations in human degenerative diseases and aging. Biochim. Biophys. Acta.

[4] Wallace DC (1997). Mitochondrial DNA in aging and disease. Sci. Am..

[5] Wallace DC (2010). Mitochondrial DNA mutations in disease and aging. Environ. Mol. Mutagen..

[6] Moraes CT, Schon EA, DiMauro S, Miranda AF (1989). Heteroplasmy of mitochondrial genomes in clonal cultures from patients with Kearns-Sayre syndrome. Biochem. Biophys. Res. Commun..

[7] Shoffner JM (1990). Myoclonic epilepsy and ragged-red fiber disease (MERRF) is associated with a mitochondrial DNA tRNA(Lys) mutation. Cell.

[8] Shoffner JM (1989). Spontaneous Kearns-Sayre/chronic external ophthalmoplegia plus syndrome associated with a mitochondrial DNA deletion: a slip-replication model and metabolic therapy. Proc. Natl Acad. Sci. USA.

[9] Wallace DC (2005). A mitochondrial paradigm of metabolic and degenerative diseases, aging, and cancer: a dawn for evolutionary medicine. Annu. Rev. Genet.

[10] Moraes CT, Atencio DP, Oca-Cossio J, Diaz F (2003). Techniques and pitfalls in the detection of pathogenic mitochondrial DNA mutations. J. Mol. Diagn..

[11] Anderson S (1981). Sequence and organization of the human mitochondrial genome. Nature.

[12] Belmonte FR (2016). Digital PCR methods improve detection sensitivity and measurement precision of low abundance mtDNA deletions. Sci. Rep..

[13] Hjelm BE (2019). Splice-Break: exploiting an RNA-seq splice junction algorithm to discover mitochondrial DNA deletion breakpoints and analyses of psychiatric disorders. Nucleic Acids Res..

[14] Bosworth CM, Grandhi S, Gould MP, LaFramboise T (2017). Detection and quantification of mitochondrial DNA deletions from next-generation sequence data. BMC Bioinform..

[15] Basu S (2020). Accurate mapping of mitochondrial DNA deletions and duplications using deep sequencing. PLoS Genet.

[16] Goudenège D (2019). eKLIPse: a sensitive tool for the detection and quantification of mitochondrial DNA deletions from next-generation sequencing data. Genet Med..

[17] Lujan SA (2020). Ultrasensitive deletion detection links mitochondrial DNA replication, disease, and aging. Genome Biol..

[18] Simchovitz A (2020). A lncRNA survey finds increases in neuroprotective LINC-PINT in Parkinson’s disease substantia nigra. Aging Cell.

[19] Nativio R (2020). An integrated multi-omics approach identifies epigenetic alterations associated with Alzheimer’s disease. Nat. Genet.

[20] Zeppillo, T. et al. Functional impairment of cortical AMPA receptors in schizophrenia. *Schizophr. Res.*10.1016/j.schres.2020.03.037 (2020). 10.1016/j.schres.2020.03.037 PMC7718399 32513544

[21] Kim S, Webster MJ (2010). The stanley neuropathology consortium integrative database: a novel, web-based tool for exploring neuropathological markers in psychiatric disorders and the biological processes associated with abnormalities of those markers. Neuropsychopharmacology.

[22] Lavin KM (2020). Rehabilitative impact of exercise training on human skeletal muscle transcriptional programs in Parkinson’s disease. Front Physiol.

[23] Tumasian RA (2021). Skeletal muscle transcriptome in healthy aging. Nat. Commun..

[24] Aguila J (2021). Spatial RNA sequencing identifies robust markers of vulnerable and resistant human midbrain dopamine neurons and their expression in Parkinson’s disease. Front. Mol. Neurosci..

[25] Monzón-Sandoval J (2020). Human-specific transcriptome of ventral and dorsal midbrain dopamine neurons. Ann. Neurol..

[26] Maynard KR (2021). Transcriptome-scale spatial gene expression in the human dorsolateral prefrontal cortex. Nat. Neurosci..

[27] Enge M (2017). Single-cell analysis of human pancreas reveals transcriptional signatures of aging and somatic mutation patterns. Cell.

[28] Darmanis S (2015). A survey of human brain transcriptome diversity at the single cell level. Proc. Natl Acad. Sci. USA.

[29] Consortium G (2013). The Genotype-Tissue Expression (GTEx) project. Nat. Genet..

[30] Nissanka N, Minczuk M, Moraes CT (2019). Mechanisms of mitochondrial DNA deletion formation. Trends Genet..

[31] Thyagarajan B, Padua RA, Campbell C (1996). Mammalian mitochondria possess homologous DNA recombination activity. J. Biol. Chem..

[32] Lakshmipathy U, Campbell C (1999). Double strand break rejoining by mammalian mitochondrial extracts. Nucleic Acids Res..

[33] Mita S (1990). Recombination via flanking direct repeats is a major cause of large-scale deletions of human mitochondrial DNA. Nucleic Acids Res..

[34] Damas J, Carneiro J, Amorim A, Pereira F (2014). MitoBreak: the mitochondrial DNA breakpoints database. Nucleic Acids Res..

[35] Schon EA (1989). A direct repeat is a hotspot for large-scale deletion of human mitochondrial DNA. Science.

[36] Zeviani M (1988). Deletions of mitochondrial DNA in Kearns-Sayre syndrome. Neurology.

[37] Moraes CT (1989). Mitochondrial DNA deletions in progressive external ophthalmoplegia and Kearns-Sayre syndrome. N Engl. J. Med..

[38] Johns DR, Threlkeld AB, Miller NR, Hurko O (1993). Multiple mitochondrial DNA deletions in myo-neuro-gastrointestinal encephalopathy syndrome. Am. J. Ophthalmol..

[39] Nakai A (1994). Diffuse leukodystrophy with a large-scale mitochondrial DNA deletion. Lancet.

[40] Moslemi AR, Melberg A, Holme E, Oldfors A (1996). Clonal expansion of mitochondrial DNA with multiple deletions in autosomal dominant progressive external ophthalmoplegia. Ann. Neurol..

[41] Paramasivam A (2016). Novel mutation in C10orf2 associated with multiple mtDNA deletions, chronic progressive external ophthalmoplegia and premature aging. Mitochondrion.

[42] Ozawa T (1990). Quantitative determination of deleted mitochondrial DNA relative to normal DNA in parkinsonian striatum by a kinetic PCR analysis. Biochem. Biophys. Res. Commun..

[43] Wei YH (1992). Mitochondrial DNA alterations as ageing-associated molecular events. Mutat. Res..

[44] Phillips NR, Simpkins JW, Roby RK (2014). Mitochondrial DNA deletions in Alzheimer’s brains: a review. Alzheimers Dement.

[45] Yan F, Powell DR, Curtis DJ, Wong NC (2020). From reads to insight: a hitchhiker’s guide to ATAC-seq data analysis. Genome Biol..

[46] Amemiya HM, Kundaje A, Boyle AP (2019). The ENCODE blacklist: identification of problematic regions of the genome. Sci. Rep..

[47] Preissl S (2018). Author Correction: Single-nucleus analysis of accessible chromatin in developing mouse forebrain reveals cell-type-specific transcriptional regulation. Nat. Neurosci..

[48] Ma S (2020). Chromatin potential identified by shared single-cell profiling of RNA and chromatin. Cell.

[49] Rickner, H. D., Niu, S. Y. & Cheng, C. S. ATAC-seq assay with low mitochondrial DNA contamination from primary human CD4+ T lymphocytes. *J. Vis. Exp.*10.3791/59120 (2019). 10.3791/59120 PMC7203994 30958473

[50] Li X, Nair A, Wang S, Wang L (2015). Quality control of RNA-seq experiments. Methods Mol. Biol..

[51] Alvarez M (2020). Enhancing droplet-based single-nucleus RNA-seq resolution using the semi-supervised machine learning classifier DIEM. Sci. Rep..

[52] Poduri A, Evrony GD, Cai X, Walsh CA (2013). Somatic mutation, genomic variation, and neurological disease. Science.

[53] Stewart JB, Chinnery PF (2015). The dynamics of mitochondrial DNA heteroplasmy: implications for human health and disease. Nat. Rev. Genet..

[54] Rollins BL (2018). Mitochondrial complex I deficiency in schizophrenia and bipolar disorder and medication influence. Mol. Neuropsychiatry.

[55] Zhang W, Cui H, Wong LJ (2012). Comprehensive one-step molecular analyses of mitochondrial genome by massively parallel sequencing. Clin. Chem..

[56] Li H (2009). The sequence alignment/map format and SAMtools. Bioinformatics.

[57] Quinlan AR, Hall IM (2010). BEDTools: a flexible suite of utilities for comparing genomic features. Bioinformatics.

[58] Kim D, Paggi JM, Park C, Bennett C, Salzberg SL (2019). Graph-based genome alignment and genotyping with HISAT2 and HISAT-genotype. Nat. Biotechnol..

[59] Martin FJ (2023). Ensembl 2023. Nucleic Acids Res..

[60] Satija R, Farrell JA, Gennert D, Schier AF, Regev A (2015). Spatial reconstruction of single-cell gene expression data. Nat. Biotechnol..

[61] Wickham, H. *ggplot2: Elegant Graphics for Data Analysis* (Springer, 2016).

[62] Gu Z, Eils R, Schlesner M (2016). Complex heatmaps reveal patterns and correlations in multidimensional genomic data. Bioinformatics.

[63] Zhou, L. et al. ggmsa: a visual exploration tool for multiple sequence alignment and associated data. *Brief Bioinform*. **23**10.1093/bib/bbac222 (2022). 10.1093/bib/bbac222 35671504

[64] Andrews RM (1999). Reanalysis and revision of the Cambridge reference sequence for human mitochondrial DNA. Nat. Genet..

[65] Hwang Y (2013). Gene expression profiling by mRNA sequencing reveals increased expression of immune/inflammation-related genes in the hippocampus of individuals with schizophrenia. Transl. Psychiatry.

[66] Prakrithi P, Juwayria, Jain D, Malik PS, Gupta I (2023). Caution towards spurious off-target signal in 10X Visium spatial transcriptomics assay from potential lncRNAs. Brief. Bioinform..

[67] Thompson LV (2006). Oxidative stress, mitochondria and mtDNA-mutator mice. Exp. Gerontol..

[68] Kujoth GC, Bradshaw PC, Haroon S, Prolla TA (2007). The role of mitochondrial DNA mutations in mammalian aging. PLoS Genet.

[69] Corral-Debrinski M (1992). Mitochondrial DNA deletions in human brain: regional variability and increase with advanced age. Nat. Genet..

[70] Cortopassi GA, Shibata D, Soong NW, Arnheim N (1992). A pattern of accumulation of a somatic deletion of mitochondrial DNA in aging human tissues. Proc. Natl Acad. Sci. USA.

[71] Copeland WC, Longley MJ (2014). Mitochondrial genome maintenance in health and disease. DNA Repair (Amst.).

[72] Cline SD (2012). Mitochondrial DNA damage and its consequences for mitochondrial gene expression. Biochim. Biophys. Acta.

[73] Kim HR (2015). Mitochondrial DNA aberrations and pathophysiological implications in hematopoietic diseases, chronic inflammatory diseases, and cancers. Ann. Lab. Med..

[74] Harris JJ, Attwell D (2012). The energetics of CNS white matter. J. Neurosci..

[75] Harris KD, Shepherd GM (2015). The neocortical circuit: themes and variations. Nat. Neurosci..

[76] Slomovic S, Laufer D, Geiger D, Schuster G (2005). Polyadenylation and degradation of human mitochondrial RNA: the prokaryotic past leaves its mark. Mol. Cell Biol..

[77] Nagaike T, Suzuki T, Ueda T (2008). Polyadenylation in mammalian mitochondria: insights from recent studies. Biochim. Biophys. Acta..

[78] Shepard PJ (2011). Complex and dynamic landscape of RNA polyadenylation revealed by PAS-Seq. RNA.

[79] Bender A (2006). High levels of mitochondrial DNA deletions in substantia nigra neurons in aging and Parkinson disease. Nat. Genet..

[80] Kraytsberg Y (2006). Mitochondrial DNA deletions are abundant and cause functional impairment in aged human substantia nigra neurons. Nat. Genet..

[81] Dölle C (2016). Defective mitochondrial DNA homeostasis in the substantia nigra in Parkinson disease. Nat. Commun..

[82] Reeve AK (2008). Nature of mitochondrial DNA deletions in substantia nigra neurons. Am. J. Hum. Genet..

[83] Mamdani F, Rollins B, Morgan L, Sequeira PA, Vawter MP (2014). The somatic common deletion in mitochondrial DNA is decreased in schizophrenia. Schizophr. Res..

[84] Laughlin SB, Sejnowski TJ (2003). Communication in neuronal networks. Science.

[85] Yu Y, Herman P, Rothman DL, Agarwal D, Hyder F (2018). Evaluating the gray and white matter energy budgets of human brain function. J Cereb. Blood Flow Metab..

[86] Campbell GR (2011). Mitochondrial DNA deletions and neurodegeneration in multiple sclerosis. Ann. Neurol..

[87] Lott MT (2013). mtDNA variation and analysis using mitomap and mitomaster. Curr. Protoc. Bioinform..

[88] Omidsalar, A. A. et al. (Zenodo, 10.5281/zenodo.10499375).

[89] Hjelm, B. E. et al. (Zenodo, 10.5281/zenodo.10499097).

