# Supplementary material for: Common mitochondrial deletions in RNA-Seq: evaluation of bulk, single-cell, and spatial transcriptomic datasets
Source: Commun Biol. 2024 Feb 17;7:200. doi: 10.1038/s42003-024-05877-4 (PMC10874445; doi:10.1038/s42003-024-05877-4)
Supplement: Supplementary file 1 — Supplementary Information [file 42003_2024_5877_MOESM1_ESM.pdf]

Brain (DLPFC) – SCZ & CTRL: DNA vs RNA

Deletion	# of Deletions	Spearman's rho	Spearman's p-value	Spearman's p-value (BH adjusted)	Pearson's correlation	Pearson's p-value	Pearson's p-value (BH adjusted)
**6335-13999**	**1**	**0.408973**	**0.02483**	**0.08513143**	**0.2349793**	**0.2113**	**0.33808**
**7816-14807**	**1**	**0.3423992**	**0.06401**	**0.162048**	**0.4659447**	**0.009457**	**0.056208**
**8471-13449**	**1**	**0.739537**	**3.03E-06**	**0.00007272**	**0.672414**	**4.70E-05**	**0.000564**
6545-13846	1	0.06526371	0.7319	0.90302609	-0.0856829	0.6526	0.71192727
7126-14004	1	0.4136976	0.02305	0.08513143	0.7353519	3.68E-06	0.00008832
7720-15821	1	NA	NA	NA	NA	NA	NA
1105-13846	1	NA	NA	NA	NA	NA	NA
6329-13994	1	NA	NA	NA	NA	NA	NA
7981-15503	1	0.3781021	0.03939	0.11817	0.2650998	0.1568	0.27188571
983-13803	1	NA	NA	NA	NA	NA	NA
7499-14426	1	-0.0323073	0.8654	0.90302609	-0.066461	0.7271	0.75871304
8471-14377	1	0.1444689	0.4463	0.66945	0.1567946	0.408	0.544
1714-15517	1	NA	NA	NA	NA	NA	NA
7863-15382	1	-0.0844391	0.6573	0.90302609	0.3146699	0.09034	0.19710545
7126-14529	1	-0.0636699	0.7382	0.90302609	-0.1071179	0.5732	0.65508571
8574-13999	1	0.2242738	0.2335	0.3736	0.3608059	0.05014	0.120336
8624-14815	1	-0.0328438	0.8632	0.90302609	-0.1144309	0.5471	0.65508571
1127-13868	1	NA	NA	NA	NA	NA	NA
5368-14055	1	0.3130416	0.09211	0.17004923	0.5111795	0.003891	0.031128
6219-13449	1	0.0359926	0.8502	0.90302609	-0.1126379	0.5534	0.65508571
7807-13999	1	NA	NA	NA	NA	NA	NA
8471-13584	1	0.3199286	0.0848	0.1696	0.2640463	0.1586	0.27188571
8624-15335	1	0.245455	0.1911	0.3276	0.2230342	0.2361	0.35415
8624-14055	1	0.4913855	0.005823	0.0279504	0.3684703	0.04512	0.12032
5368-15335	1	NA	NA	NA	NA	NA	NA
6545-14419	1	NA	NA	NA	NA	NA	NA
6840-11136	1	0.3231266	0.08156	0.1696	0.2859551	0.1256	0.2512
7863-13449	1	0.05657518	0.7665	0.90302609	-0.028324	0.8819	0.8819
7816-15382	1	0.3382392	0.06752	0.162048	0.4125602	0.02347	0.07041
3789-14807	1	NA	NA	NA	NA	NA	NA
Top 10 Cumulative Deletions	10	0.5288098	0.003044	0.018264	0.4127966	0.02339	0.07041
Top 20 Cumulative Deletions	20	0.5661846	0.001338	0.010704	0.4337123	0.01665	0.0666
**Top 30 Cumulative Deletions**	**30**	**0.5671376**	**0.001083**	**0.010704**	**0.454089**	**0.01171**	**0.056208**
High Frequency Deletions	112	-0.0002224694	1	1	0.1769713	0.3495	0.49341176

Supplementary Table 1. Correlations of high frequency mtDNA deletions in DNA-Seq and RNA-Seq. RNA-Seq data from the brain[20] was processed through the Splice-Break2 pipeline and compared to results using the traditional mtDNA enrichment and DNA-Sequencing approach[13,55]. Pearson's and Spearman's correlations between DNA-Seq and RNA-Seq data are shown comparing each of the "Top 30" most frequent deletions according to our previous study; the Top 10, Top 20, and Top 30 cumulative deletions; and all the deletions of high frequency from our previous study[13]. P-values are shown with and without Benjamini-Hochberg (BH) corrections for multiple comparisons.

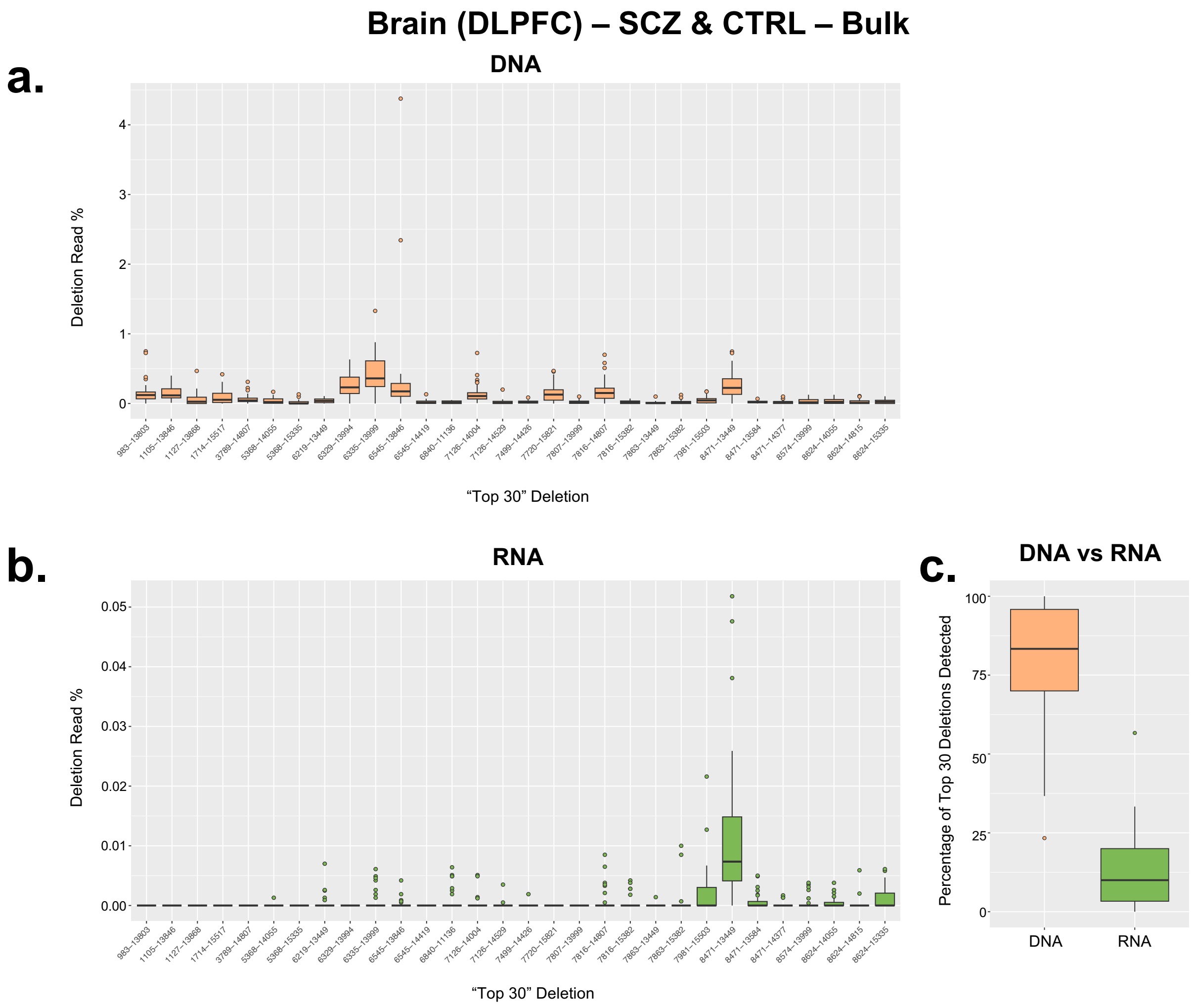

Supplementary Figure 1. Summary of "Top 30" mtDNA deletions.
Boxplots showing deletion read % of each of the individual "Top 30" mtDNA deletions in corresponding **(a)** DNA-Seq and **(b)** RNA-Seq data (n=30)[20]. **(c)** Percentage of the "Top 30" deletions detected in each sequencing type. All boxplots show the median as a solid black line; the first and third quartiles are captured by the bounds of the box. Boxplot whiskers are defined as the first and third quartiles ± interquartile range times 1.5, respectively, and outliers are denoted as points.

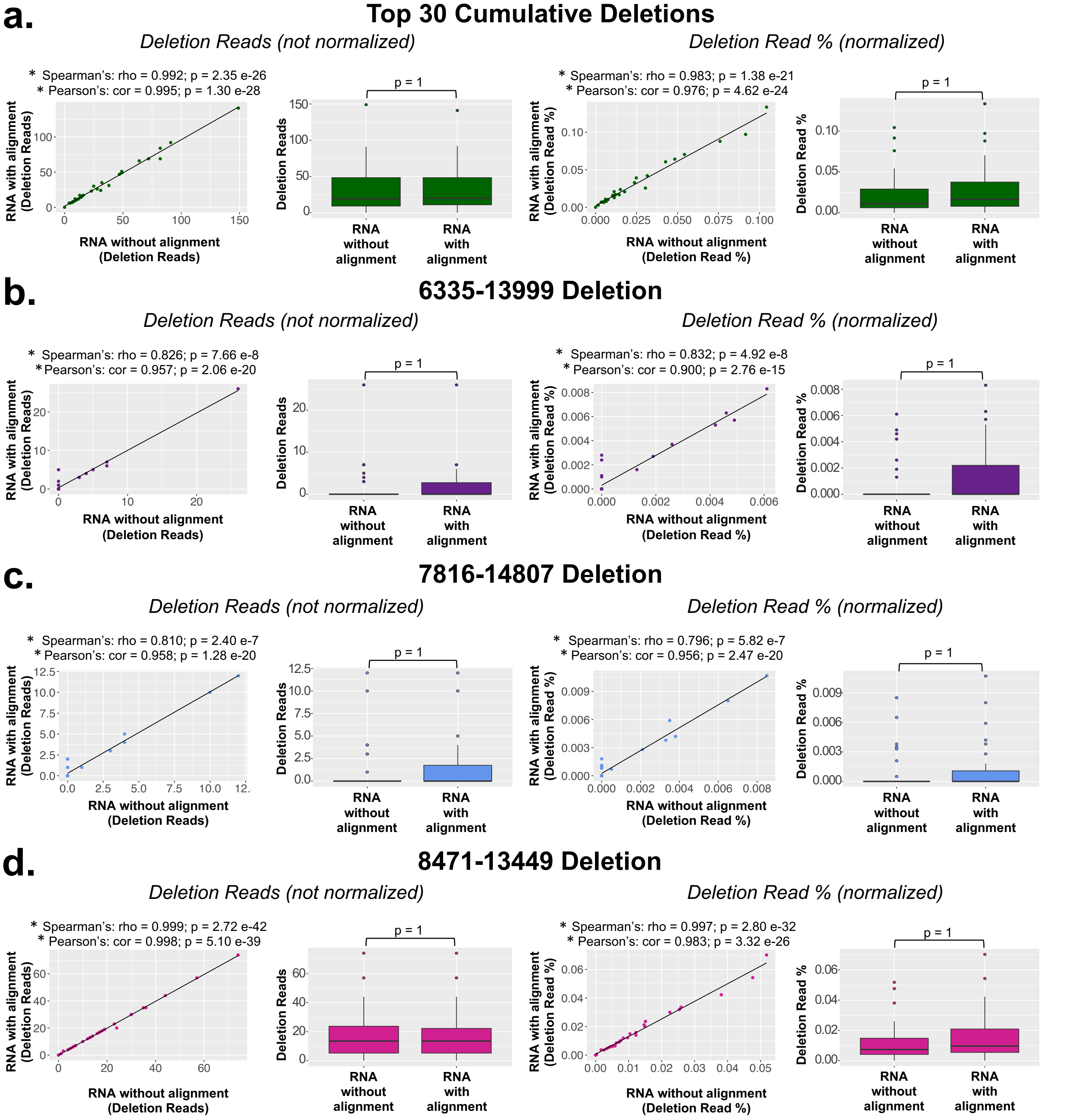

Supplementary Figure 2. Correlations and comparisons of mtDNA deletions captured by all RNA-Seq reads vs uniquely mapped RNA-Seq reads. RNA-Seq data from the brain (n=30)[20] was aligned to the nuclear genome using HISAT2; unmapped reads were processed through the Splice-Break2 pipeline and compared to results from Splice-Break2 analysis of all reads. All analyses included correlations and relative abundance of deletion reads detected in each sample (not normalized; left) and the deletion read rate for each sample (normalized; right). **(a)** The sum of the Top 30 deletions. The three most common deletions: **(b)** 6335-13999, **(c)** 7816-14807, and **(d)** 8471-13449. Spearman and Pearson's correlations are shown. Statistical values for box plots are from Welch's *t*-tests. All *p*-values were corrected for multiple tests using Bonferroni. All boxplots show the median as a solid black line; the first and third quartiles are captured by the bounds of the box. Boxplot whiskers are defined as the first and third quartiles ± interquartile range times 1.5, respectively, and outliers are denoted as points.

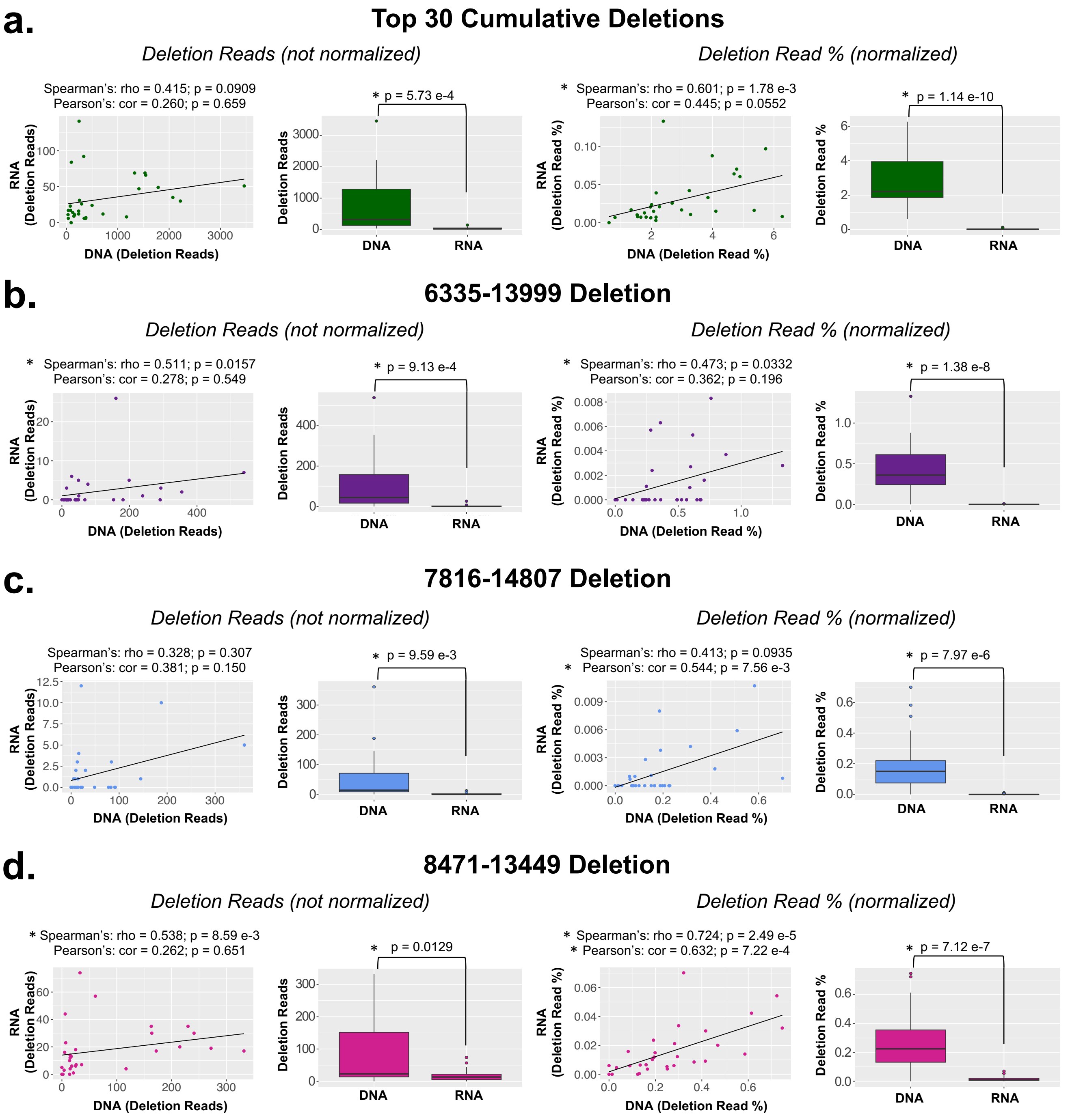

Supplementary Figure 3. Correlations and comparisons of mtDNA deletions captured by mtDNA-Seq vs uniquely mapped RNA-Seq reads. RNA-Seq data from the brain (n=30)[20] was aligned to the nuclear genome using HISAT2; unmapped reads were processed through the Splice-Break2 pipeline and compared to results using the traditional mtDNA-enrichment and DNA sequencing approach[13,55]. All analyses included correlations and relative abundance of deletion reads detected in each sample (not normalized; left) and the deletion read rate for each sample (normalized; right). **(a)** The sum of the Top 30 deletions. The three most common deletions: **(b)** 6335-13999, **(c)** 7816-14807, and **(d)** 8471-13449. Spearman and Pearson's correlations are shown. Statistical values for box plots are from Welch's *t*-tests. All *p*-values were corrected for multiple tests using Bonferroni. All boxplots show the median as a solid black line; the first and third quartiles are captured by the bounds of the box. Boxplot whiskers are defined as the first and third quartiles ± interquartile range times 1.5, respectively, and outliers are denoted as points.

a. **Brain (DLPFC) – SCZ & CTRL – Bulk**

Deletion	Females Mean ± SD	Males Mean ± SD	p-value
**6335-13999**	0.00098 ± 0.00219	0.000828 ± 0.00172	1
**7816-14807**	0.00108 ± 0.00154	0.000912 ± 0.00224	1
**8471-13449**	0.0241 ± 0.0236	0.00988 ± 0.00957	0.5868
**Top 30 Cumulative Deletions**	0.0443 ± 0.0435	0.0188 ± 0.0212	1

b. **Muscle (Skeletal) – PD & CTRL – Bulk**

Deletion	Females Mean ± SD	Males Mean ± SD	p-value
**6335-13999**	0.0088 ± 0.0227	0.0120 ± 0.0426	1
**7816-14807**	0	0.00593 ± 0.0117	0.9576
**8471-13449**	0.00563 ± 0.00955	0.0585 ± 0.137	1
**Top 30 Cumulative Deletions**	0.0305 ± 0.0294	0.109 ± 0.180	0.7138

c. **Muscle (Skeletal) – CTRL – Bulk**

Deletion	Females Mean ± SD	Males Mean ± SD	p-value
**6335-13999**	0.00446 ± 0.00821	0.00218 ± 0.00337	1
**7816-14807**	0.00298 ± 0.00831	0.00700 ± 0.0230	1
**8471-13449**	0.0110 ± 0.0237	0.00747 ± 0.0115	1
**Top 30 Cumulative Deletions**	0.0960 ± 0.183	0.103 ± 0.170	1

d. **Brain (Cerebellum) – BD, SCZ, MDD & CTRL – Bulk**

Deletion	Females Mean ± SD	Males Mean ± SD	p-value
**6335-13999**	0.000523 ± 0.00141	0.0005 ± 0.00119	1
**7816-14807**	0.00439 ± 0.00676	0.00230 ± 0.00570	0.4384
**8471-13449**	0.0015 ± 0.00320	0.000836 ± 0.00118	0.8932
**Top 30 Cumulative Deletions**	0.0192 ± 0.0244	0.0108 ± 0.0116	0.1712

e. **Brain (Hippocampus) – BD, SCZ, MDD & CTRL – Bulk**

Deletion	Females Mean ± SD	Males Mean ± SD	p-value
**6335-13999**	0.00216 ± 0.00514	0.00389 ± 0.00641	1
**7816-14807**	0.00711 ± 0.00857	0.00643 ± 0.00888	1
**8471-13449**	0.0108 ± 0.0172	0.00785 ± 0.0107	1
**Top 30 Cumulative Deletions**	0.0854 ± 0.0617	0.0804 ± 0.0619	1

f. **Brain (Prefrontal Cortex) – BD, SCZ, MDD & CTRL – Bulk**

Deletion	Females Mean ± SD	Males Mean ± SD	p-value
**6335-13999**	0.00179 ± 0.00564	0.000146 ± 0.000862	0.508
**7816-14807**	0.0000696 ± 0.000334	0	0.504
**8471-13449**	0.00749 ± 0.0111	0.00266 ± 0.00609	0.4174
**Top 30 Cumulative Deletions**	0.0179 ± 0.0188	0.00709 ± 0.0154	0.1379

Supplementary Table 2. Analyses of biological sex in GEO+ datasets. (a-e) Average expression (mean ± SD) by biological sex of the 6335-13999 deletion, 7816-14807 deletion, 8471-13449 deletion, and Top 30 cumulative deletions in brain[20,21] and skeletal muscle[22,23]. **(a)** DLPFC samples (n=29)[20], with one single female outlier removed from analysis; **(b)** skeletal muscle samples (n=36)[22]; **(c)** skeletal muscle samples (n=30)[23]. **(d-f)** Stanley Neuropathy Consortium data[21]: **(d)** cerebellum samples (n=58)[21]; **(e)** hippocampus samples (n=58)[21]; **(f)** prefrontal cortex samples (n=58)[21]. *P*-values shown are from linear regression models for Deletion ~ Sex and include MT benchmark coverage, age, and diagnosis as co-variates. All *p*-values were corrected for multiple tests using Bonferroni.

Muscle (Skeletal) – Aging – Bulk

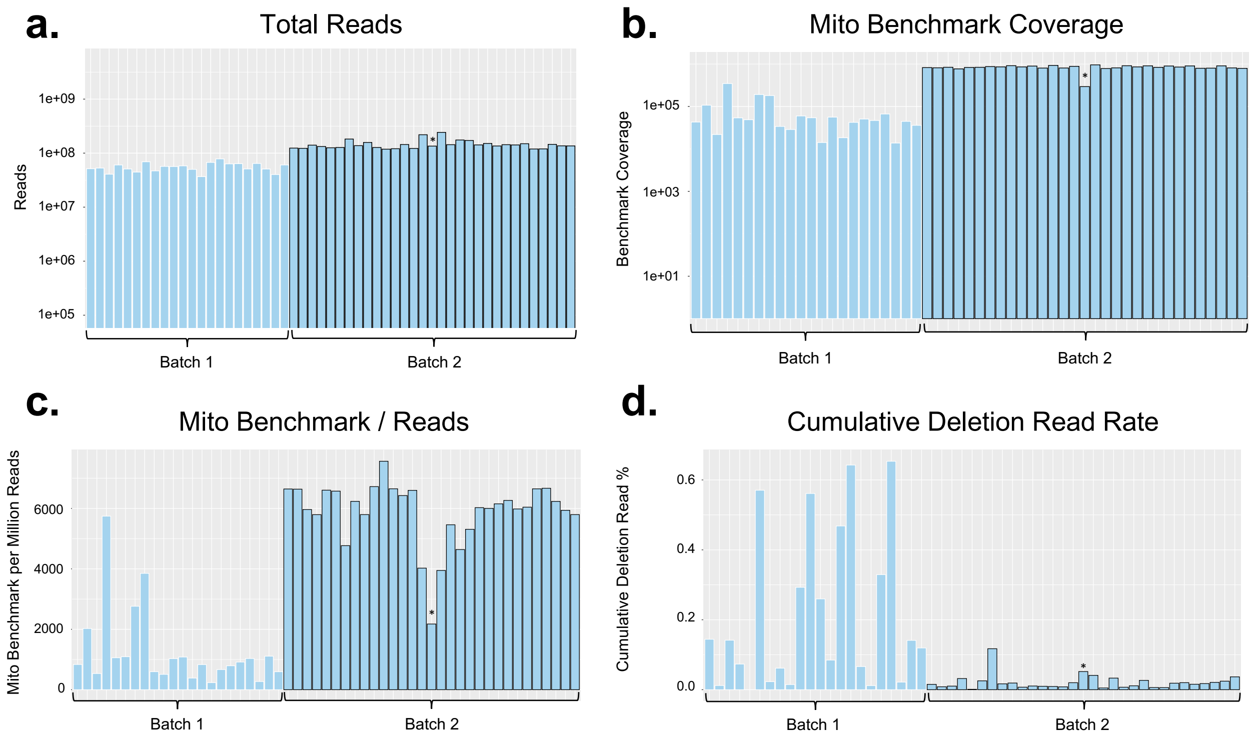

Supplementary Figure 4. Sequencing metrics of the bulk sequencing skeletal muscle control dataset[23]. A batch effect was observed in the amount of mitochondrial data (i.e., the dichotomy in initial reads, MT benchmark coverage, and MT benchmark per million reds). The first 22 SRR IDs had considerably less mitochondrial data than the last 31 SRR IDs. As such, we included the subset or “batch” of samples with more mitochondrial data (Batch 2; GSE164471: SRR13388754-SRR13388784; n=30) in our age analysis (see Fig. 4C). **(a)** The total number of FASTQ reads prior to alignment. **(b)** MT benchmark coverage (the average mitochondrial sequencing depth measured from two 250bp segments within the RNR1 and CYB genes)[13]. **(c)** MT benchmark coverage per million RNA-Seq reads. **(d)** Cumulative deletion read % (deletion reads / MT Benchmark Coverage) for the Top 30 mitochondrial deletion sum. Sample from batch 2 with asterisk (*) was an outlier removed from aging analysis.

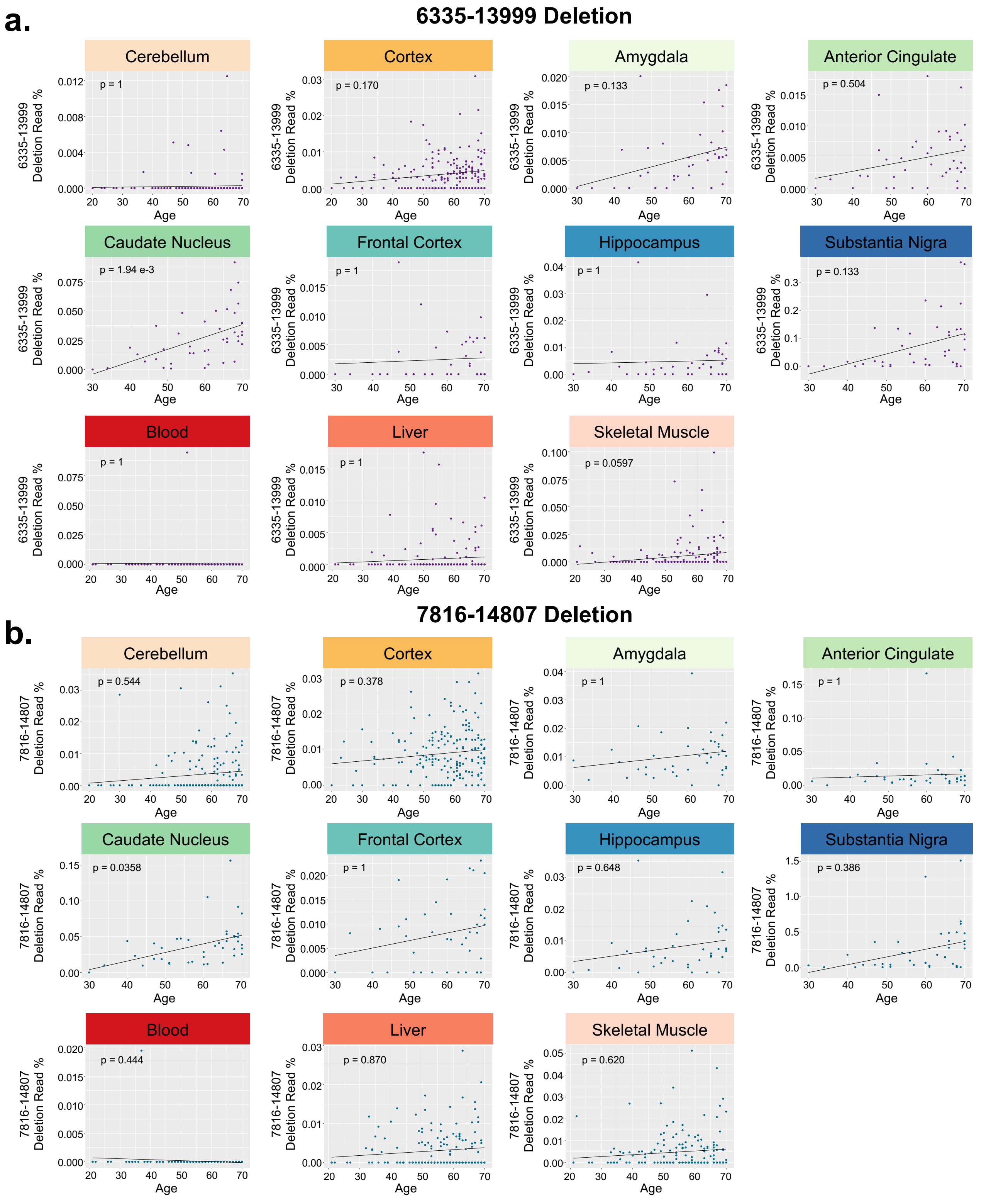

Supplementary Figure 5. Analyses of age in GTEx samples. Correlations between the 6335-13999 deletion and 7816-14807 deletion read rates with age in 11 GTEx tissues[29]. The deletion read rate of the **(a)** 6335-13999 deletion and **(b)** the 7816-14807 deletion. Tissues are from three paired datasets: 183 paired samples of cerebellum and cortex, 41 paired samples from multiple brain regions (i.e., amygdala, anterior cingulate cortex, caudate nucleus, frontal cortex, hippocampus, and substantia nigra), and 165 paired samples from non-brain regions (i.e., blood, liver, and skeletal muscle). *P*-values shown from linear regression models for Deletion ~ Age, and include MT benchmark coverage and sex as co-variates. All *p*-values were corrected for multiple tests using Bonferroni.

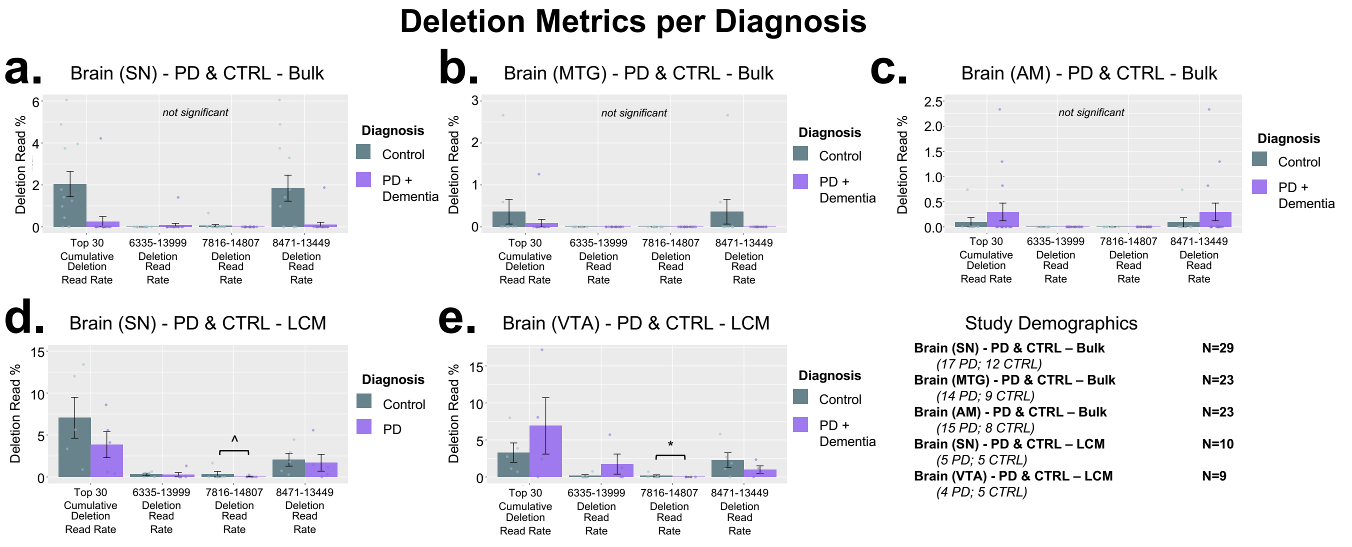

Supplementary Figure 6. Analyses of diagnosis in various brain regions in GEO+ samples.
(a-e) Comparisons of diagnosis for the “Top 30” cumulative deletions, 6335-13999, and 8471-13449 deletion in two studies[18,24]. Bar graphs represent mean ± SEM. Sample size per tissue/diagnosis: **(a)** PD+Dementia (n=17), CTRL (n=12)[18]; **(b)** PD+Dementia (n=14), CTRL (n=9)[18]; **(c)** PD+Dementia (n=15), CTRL (n=8)[18]; **(d)** PD (n=5), CTRL (n=5)[24]; **(e)** PD (n=4), CTRL (n=5)[24]. **(a)** and **(d)** are also shown in Fig. 6. *P*-values shown from linear regression models for Deletion ~ Diagnosis and include MT benchmark coverage, age, and sex as co-variates. All *p*-values were corrected for multiple tests using Bonferroni. Abbreviations: MTG (middle temporal gyrus); AM (amygdala); SN (substantia nigra); VTA (ventral tegmental area). Symbols: ^ ($p<0.05$); * ($p<0.01$).

a.

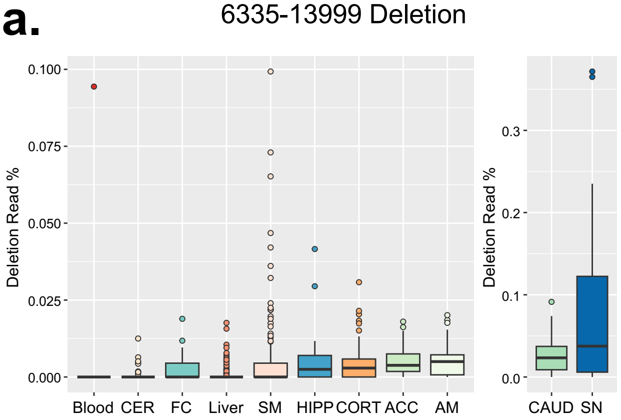

6335-13999	Blood	CER	FC	Liver	SM	HIPP	CORT	ACC	AM	CAUD	SN
Blood	1	1	0.107	1	**7.58E-04**	**0.0118**	**5.39E-06**	**3.06E-05**	**4.14E-05**	**8.49E-09**	**3.56E-05**
CER		1	**3.89E-03**	**0.0144**	**2.35E-05**	**2.68E-03**	**1.86E-19**	**2.89E-07**	**1.45E-06**	**6.19E-09**	**3.29E-05**
FC			1	0.0709	0.153	0.391	0.227	0.0572	**0.0303**	**5.42E-08**	**5.38E-05**
Liver				1	**3.84E-04**	**0.0122**	**2.20E-12**	**7.02E-06**	**1.96E-05**	**1.12E-08**	**3.78E-05**
SM					1	1	1	1	1	**7.19E-07**	**9.26E-05**
HIPP						1	1	1	1	**7.93E-07**	**9.09E-05**
CORT							1	0.884	0.425	**2.01E-07**	**7.23E-05**
ACC								1	1	**5.56E-07**	**8.94E-05**
AM									1	**1.01E-06**	**1.01E-04**
CAUD										1	**8.50E-03**
SN											1

b.

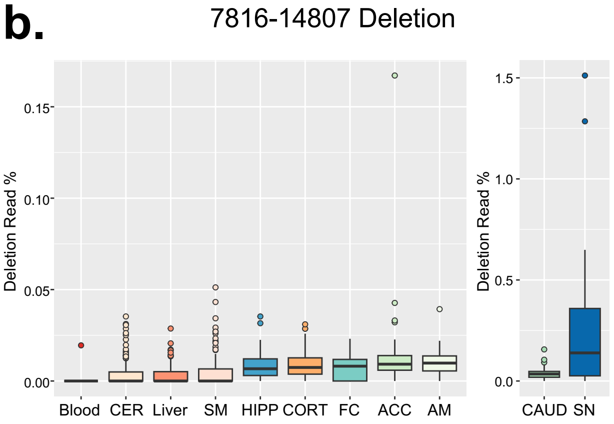

7816-14807	Blood	CER	Liver	SM	HIPP	CORT	FC	ACC	AM	CAUD	SN
Blood	1	**1.82E-09**	**1.88E-11**	**6.58E-11**	**4.55E-07**	**2.74E-40**	**4.20E-08**	**2.39E-03**	**2.66E-10**	**7.90E-10**	**1.15E-04**
CER		1	1	0.384	**3.18E-03**	**7.40E-13**	**0.00164**	**0.0251**	**5.00E-06**	**7.69E-09**	**1.41E-04**
Liver			1	0.0675	**9.15E-04**	**7.87E-18**	**3.50E-04**	**0.0186**	**1.02E-06**	**5.71E-09**	**1.37E-04**
SM				1	0.0663	**4.64E-06**	0.0613	0.0602	**3.77E-04**	**1.99E-08**	**1.53E-04**
HIPP					1	1	1	0.448	0.996	**2.84E-07**	**1.90E-04**
CORT						1	1	0.507	0.978	**3.44E-07**	**1.96E-04**
FC							1	0.364	0.566	**2.04E-07**	**1.86E-04**
ACC								1	1	**9.69E-04**	**2.96E-04**
AM									1	**1.17E-06**	**2.15E-04**
CAUD										1	**1.21E-03**
SN											1

Supplementary Figure 7. Analyses of brain region and tissue in GTEx samples. (a-b) Boxplots showing comparisons across all 11 GTEx tissues and/or brain regions[29]. **(a)** The 6335-13999 deletion for all GTEx tissues and matrix of of *p*-values for individual comparisons. **(b)** The 7816-14807 deletion for all GTEx tissues and matrix of of *p*-values for individual comparisons. Statistical values are from Welch's *t*-tests. All p-values were corrected for multiple tests using Bonferroni. Sample size per tissue: cerebellum and cortex (n=183 ea.), amygdala, anterior cingulate cortex, caudate nucleus, frontal cortex, hippocampus, and substantia nigra (n=41 ea.); blood, liver, and skeletal muscle (n=165 ea.). Abbreviations: CER (cerebellum); CORT (cortex); FC (frontal cortex); HIPP (hippocampus); ACC (anterior cingulate cortex); AM (amygdala); CAUD (caudate nucleus); SN (substantia nigra); SM (skeletal muscle). All boxplots show the median as a solid black line; the first and third quartiles are captured by the bounds of the box. Boxplot whiskers are defined as the first and third quartiles ± interquartile range times 1.5, respectively, and outliers are denoted as points.

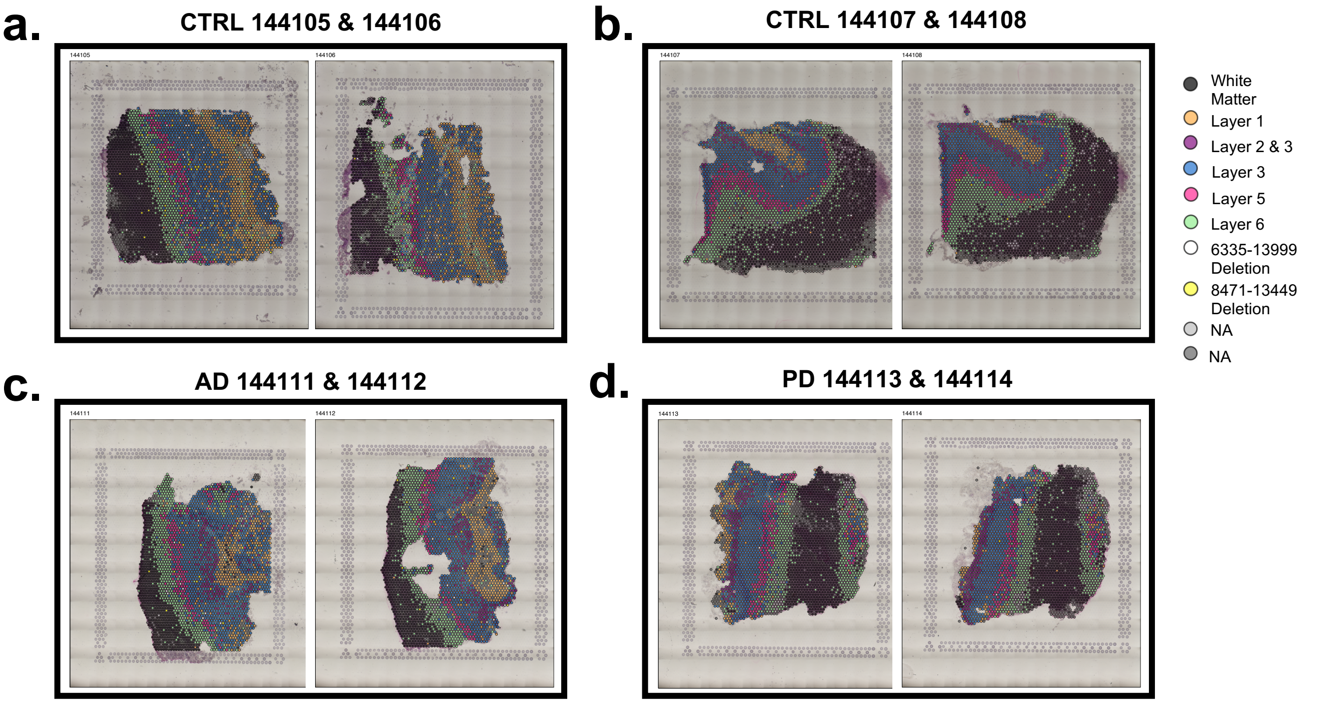

Supplementary Figure 8. Mitochondrial deletions in spatial transcriptomics and in cortical layers. **(a-d)** Spatial images of 8x USC MTG samples, colored by imputed cortical layer and spots containing 6335-13999 or 8471-13449 deletions.

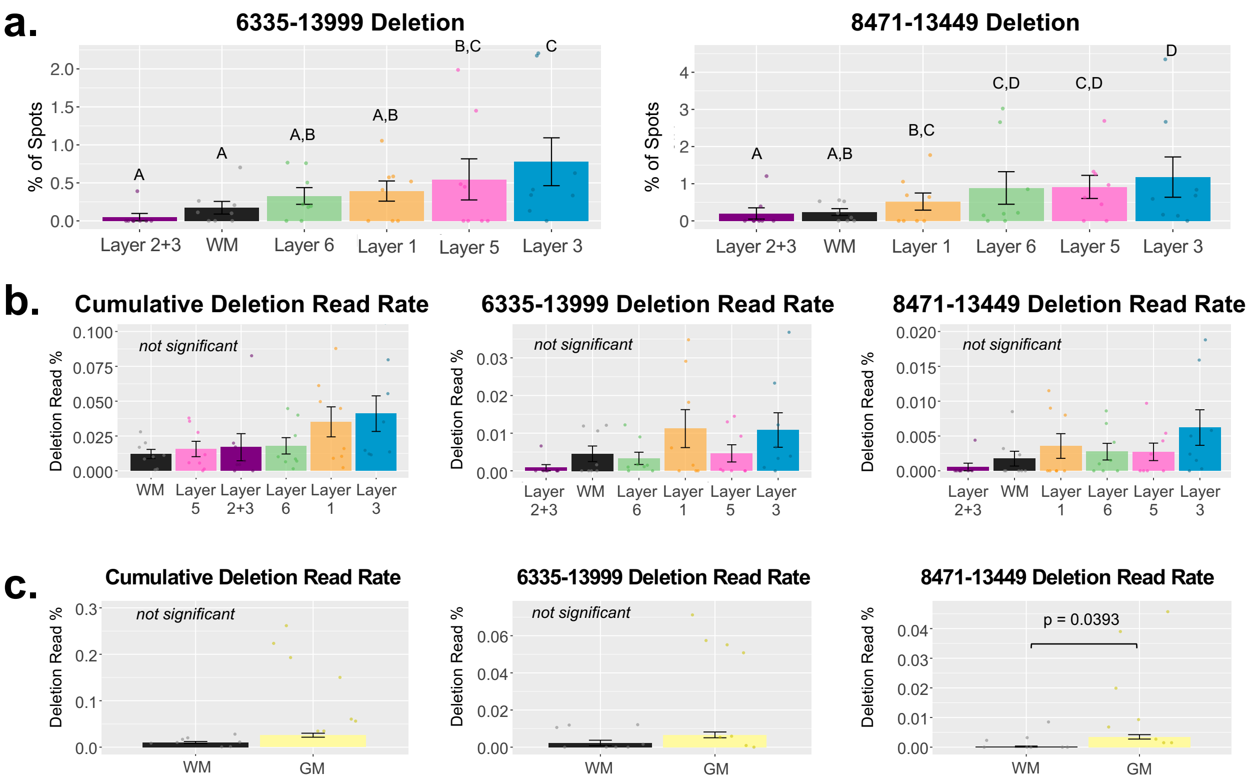

Supplementary Figure 9. Mitochondrial deletions in spatial transcriptomics and in cortical layers.
(a) Percentage of spots (mean ± SEM) from each imputed layer that contained 6335-13999 (left) or 8471-13449 (right) deletions. All 8x USC MTG samples were analyzed together. Letters above graphs describe significant differences between cortical layers from two-proportion Z-tests. Layers represented by different letters: $p<0.05$. **(b-c)** Relative abundance (average deletion read % ± SEM) of "Top 30" cumulative deletions (left), 6335-13999 deletion (middle), and 8471-13449 deletion (right) in **(b)** each imputed layer and **(c)** between grey and white matter. Layer comparisons in **(b)** are from ANOVA with MT benchmark coverage as a co-variate. Statistical values in **(c)** are from linear regression models for Deletion ~ WM/GM with MT benchmark coverage, diagnosis, age, and sex as co-variates; *p*-values were corrected for multiple tests using Bonferroni.
