# Supplementary material for: Common mitochondrial deletions in RNA-Seq: evaluation of bulk, single-cell, and spatial transcriptomic datasets
Source: Commun Biol. 2024 Feb 17;7:200. doi: 10.1038/s42003-024-05877-4 (PMC10874445; doi:10.1038/s42003-024-05877-4)
Supplement: Supplementary file 5 — Reporting Summary [file 42003_2024_5877_MOESM5_ESM.pdf]

nature portfolio

Corresponding author(s): Brooke E. Hjelm, PhD

Last updated by author(s): Nov 15, 2023

Reporting Summary

Nature Portfolio wishes to improve the reproducibility of the work that we publish. This form provides structure for consistency and transparency in reporting. For further information on Nature Portfolio policies, see our Editorial Policies and the Editorial Policy Checklist.

Statistics

For all statistical analyses, confirm that the following items are present in the figure legend, table legend, main text, or Methods section.

n/a	Confirmed	
☐	☒	The exact sample size (*n*) for each experimental group/condition, given as a discrete number and unit of measurement
☐	☒	A statement on whether measurements were taken from distinct samples or whether the same sample was measured repeatedly
☐	☒	The statistical test(s) used AND whether they are one- or two-sided<br>*Only common tests should be described solely by name; describe more complex techniques in the Methods section.*
☐	☒	A description of all covariates tested
☐	☒	A description of any assumptions or corrections, such as tests of normality and adjustment for multiple comparisons
☐	☒	A full description of the statistical parameters including central tendency (e.g. means) or other basic estimates (e.g. regression coefficient) AND variation (e.g. standard deviation) or associated estimates of uncertainty (e.g. confidence intervals)
☐	☒	For null hypothesis testing, the test statistic (e.g. *F*, *t*, *r*) with confidence intervals, effect sizes, degrees of freedom and *P* value noted<br>*Give P values as exact values whenever suitable.*
☒	☐	For Bayesian analysis, information on the choice of priors and Markov chain Monte Carlo settings
☒	☐	For hierarchical and complex designs, identification of the appropriate level for tests and full reporting of outcomes
☐	☒	Estimates of effect sizes (e.g. Cohen's *d*, Pearson's *r*), indicating how they were calculated

Our web collection on statistics for biologists contains articles on many of the points above.

Software and code

Policy information about availability of computer code

Data collection	NCBI SRA toolkit (version 2.10.9) command "fastq-dump" was used to download files from publicly available repositories. See Methods section ("Downloading Files" subsection) for additional information.
Data analysis	Custom code: Splice-Break2 (https://github.com/brookehjelm/Splice-Break2). All other custom code is available at https://github.com/aomidsalar/RNA-Seq_Splice-Break2

For manuscripts utilizing custom algorithms or software that are central to the research but not yet described in published literature, software must be made available to editors and reviewers. We strongly encourage code deposition in a community repository (e.g. GitHub). See the Nature Portfolio guidelines for submitting code & software for further information.

Data

Policy information about availability of data

All manuscripts must include a data availability statement. This statement should provide the following information, where applicable:

- Accession codes, unique identifiers, or web links for publicly available datasets
- A description of any restrictions on data availability
- For clinical datasets or third party data, please ensure that the statement adheres to our policy

RNA-Seq data newly presented in this study were deposited into the Gene Expression Omnibus database under accession number GSE226663. Accession information for all datasets used in this study can be found on the GitHub page for this project (https://github.com/aomidsalar/RNA-Seq_Splice-Break2) under

"Data_Availability_and_Accession".
Accession numbers are also available in Supplementary Data Files 1 and 2

Research involving human participants, their data, or biological material

Policy information about studies with human participants or human data. See also policy information about sex, gender (identity/presentation), and sexual orientation and race, ethnicity and racism.

Reporting on sex and gender	All descriptions here refer to sex as a biological attribute and all studies used contain both sexes as defined in their corresponding GEO+ summary file.
Reporting on race, ethnicity, or other socially relevant groupings	Race, ethnicity, or other socially relevant groupings were not part of our analysis.
Population characteristics	Relevant demographic characteristics of age and diagnosis were also pulled for all samples from their corresponding summary file.
Recruitment	As all tissue for internal datasets obtained was postmortem, this does not qualify as human subjects research. The other datasets were publicly available on online repositories (GEO, GTEx, Stanley Neuropathology Consortium).
Ethics oversight	N/A

Note that full information on the approval of the study protocol must also be provided in the manuscript.

Field-specific reporting

Please select the one below that is the best fit for your research. If you are not sure, read the appropriate sections before making your selection.

☒ Life sciences ☐ Behavioural & social sciences ☐ Ecological, evolutionary & environmental sciences

For a reference copy of the document with all sections, see nature.com/documents/nr-reporting-summary-flat.pdf

Life sciences study design

All studies must disclose on these points even when the disclosure is negative.

Sample size	As this study primarily evaluated publicly available datasets, sample size for each was pre-determined based on the authors' initial investigations.
Data exclusions	Analysis of sex in Supplementary Table 2: one female sample was removed because it was a significant outlier. This is mentioned in the table legend.<br>Analysis of age in Fig. 4C: only 30/53 samples from the Tumasian et al. study were used for analysis of age because a batch effect was observed. This is described in Supplemental Figure 4 and our Results section.<br>No additional data was excluded.
Replication	We observed replicable findings for the effect of age and high levels of mtDNA deletions in dopaminergic brain regions in multiple studies. However, since these datasets were publicly available and we did not have access to the tissue, we did not replicate these findings by other experimental methods.
Randomization	This is not applicable as this study was observational and did not include an intervention.
Blinding	This is not applicable as this study was observational and bioinformatics pipeline is not influenced by a priori knowledge of subject demographics

Reporting for specific materials, systems and methods

We require information from authors about some types of materials, experimental systems and methods used in many studies. Here, indicate whether each material, system or method listed is relevant to your study. If you are not sure if a list item applies to your research, read the appropriate section before selecting a response.

Materials & experimental systems

n/a	Involved in the study
☒	☐ Antibodies
☒	☐ Eukaryotic cell lines
☒	☐ Palaeontology and archaeology
☒	☐ Animals and other organisms
☒	☐ Clinical data
☒	☐ Dual use research of concern
☒	☐ Plants

Methods

n/a	Involved in the study
☒	☐ ChIP-seq
☒	☐ Flow cytometry
☒	☐ MRI-based neuroimaging
